# Author Correction: Subclonal mutation selection in mouse lymphomagenesis identifies known cancer loci and suggests novel candidates

**DOI:** 10.1038/s41467-019-09109-w

**Published:** 2019-03-06

**Authors:** Philip Webster, Joanna C. Dawes, Hamlata Dewchand, Katalin Takacs, Barbara Iadarola, Bruce J. Bolt, Juan J. Caceres, Jakub Kaczor, Gopuraja Dharmalingam, Marian Dore, Laurence Game, Thomas Adejumo, James Elliott, Kikkeri Naresh, Mohammad Karimi, Katerina Rekopoulou, Ge Tan, Alberto Paccanaro, Anthony G. Uren

**Affiliations:** 1 MRC London Institute of Medical Sciences (LMS), Du Cane Road, London, W12 0NN UK; 2 Institute of Clinical Sciences (ICS), Faculty of Medicine, Imperial College London, Du Cane Road, London, W12 0NN UK; 3 Imperial College Healthcare NHS Trust, London, W12 0HS UK; 4 Centre for Systems and Synthetic Biology, Department of Computer Science, Royal Holloway, University of London, TW20 0EX Egham, UK

Correction to: *Nature Communications* 10.1038/s41467-018-05069-9; published online 09 July 2018

The original version of this Article contained an error in the hyperlink for the online repository http://mulvdb.org which was incorrectly given as http://mulv.lms.mrc.ac.uk. This has been corrected in both the PDF and HTML versions of the Article.

