# Supplementary material for: Subclonal mutation selection in mouse lymphomagenesis identifies known cancer loci and suggests novel candidates
Source: Nat Commun. 2018 Jul 9;9:2649. doi: 10.1038/s41467-018-05069-9 (PMC6037733; doi:10.1038/s41467-018-05069-9)
Supplement: Supplementary file 1 — Supplementary Information [file 41467_2018_5069_MOESM1_ESM.pdf]

Subclonal Mutation Selection in Mouse Lymphomagenesis Identifies Known Cancer Loci And Suggests Novel Candidates.

Webster et al.

Supplementary Figures and Tables

a

b

c

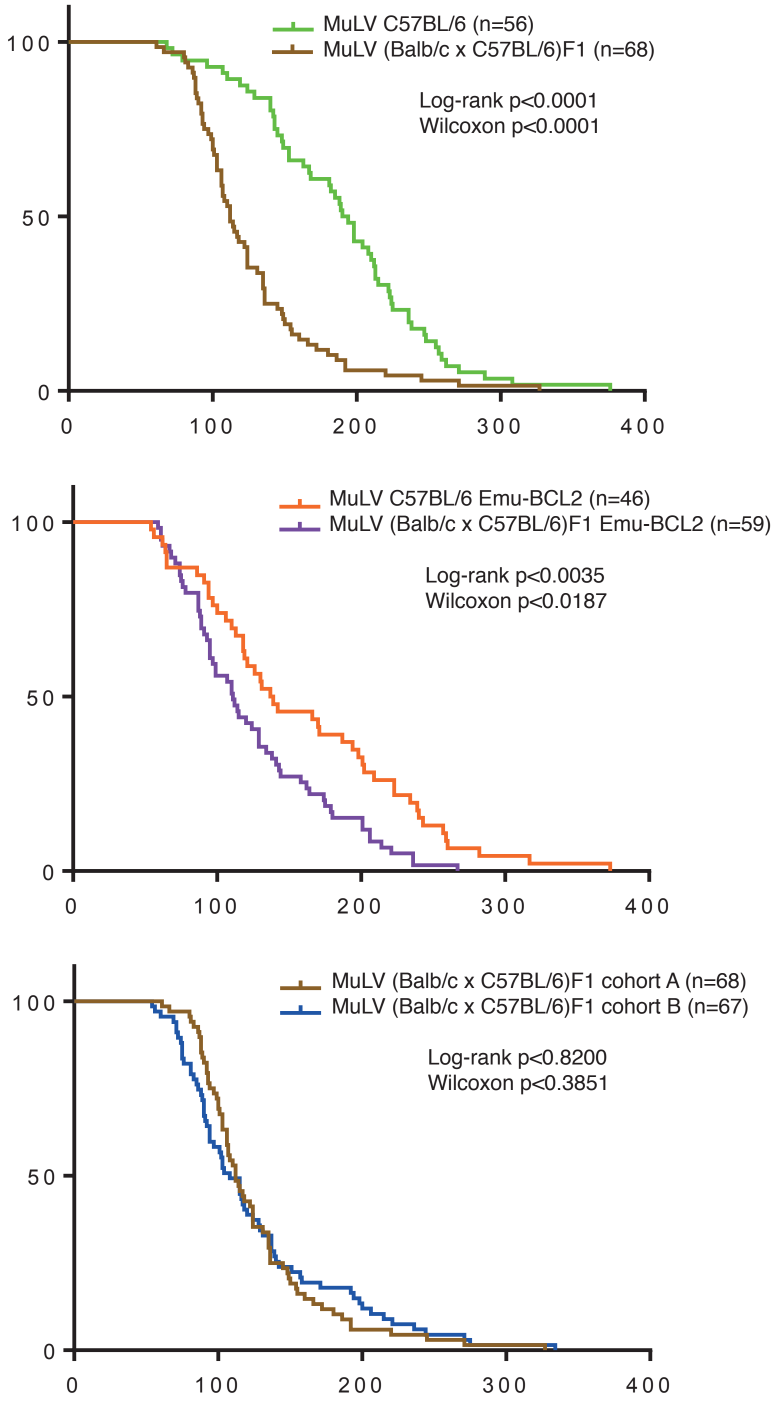
MuLV C57BL/6 (n=56)
MuLV (Balb/c x C57BL/6)F1 (n=68)
Log-rank p<0.0001
Wilcoxon p<0.0001
100
50
0
0
100
200
300
400
MuLV C57BL/6 Emu-BCL2 (n=46)
MuLV (Balb/c x C57BL/6)F1 Emu-BCL2 (n=59)
Log-rank p<0.0035
Wilcoxon p<0.0187
MuLV (Balb/c x C57BL/6)F1 cohort A (n=68)
MuLV (Balb/c x C57BL/6)F1 cohort B (n=67)
Log-rank p<0.8200
Wilcoxon p<0.3851

Supplementary Figure 1 – Cohort comparisons

Both wild type a) and Emu-BCL2 mice b) developed lymphoma significantly more rapidly on an F1 background by either the Log-rank or Wilcoxon text c) No difference in latency was observed between both cohorts of F1 wild type control animals that were generated independently.

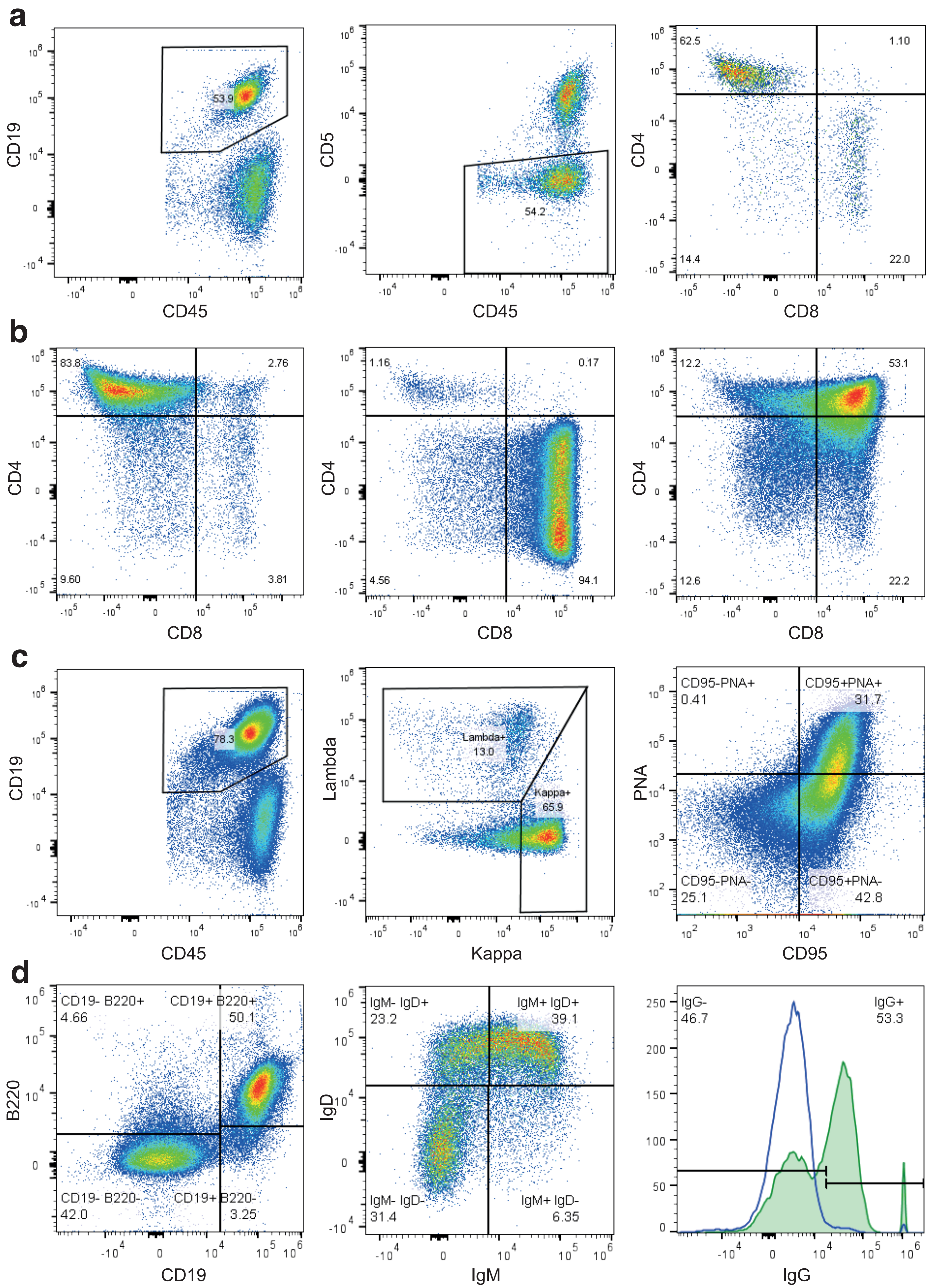

a
CD19
CD45
53.9
CD5
54.2
CD4
CD8
62.5
1.10
14.4
22.0
b
83.8
2.76
9.60
3.81
1.16
0.17
4.56
94.1
12.2
53.1
12.6
22.2
c
78.3
Lambda
Kappa
Lambda+
13.0
Kappa+
65.9
PNA
CD95
CD95-PNA+
0.41
CD95+PNA+
31.7
CD95-PNA-
25.1
CD95+PNA-
42.8
d
B220
CD19
CD19- B220+
4.66
CD19+ B220+
50.1
CD19- B220-
42.0
CD19+ B220-
3.25
IgD
IgM
IgM- IgD+
23.2
IgM+ IgD+
39.1
IgM- IgD-
31.4
IgM+ IgD-
6.35
IgG
IgG-
46.7
IgG+
53.3

Supplementary Figure 2 - Representative flow cytometry of spleen suspensions of MuLV infected animals.

a) Spleen suspensions were subjected to flow cytometry with a panel of B/T lineage markers (CD45, CD19, CD3, CD5, CD4, CD8). CD45+ cells (lymphocytes) were gated on CD19 (B cells) and CD19- CD5- cells were then gated out. The remaining CD19- CD5+ cells were gated for CD4 & CD8 staining to indicate T cell subsets. b) Representative plots of CD5+ T cell lymphomas (CD4+, CD8+ or CD4+CD8+). c) BCL2 transgenic animals typically displayed a higher proportion of CD19+ cells, with nearly all staining positive for surface kappa/lambda light chain indicating a mature B cell phenotype. Vav-BCL2 transgenic suspensions expressed a high proportion of germinal center markers CD95 and peanut agglutinin. d) CD19+ B220+ cells from Vav-BCL2 transgenic animals typically displayed a high proportion of IgM- IgD- cells that were frequently class switched IgG+ cells.

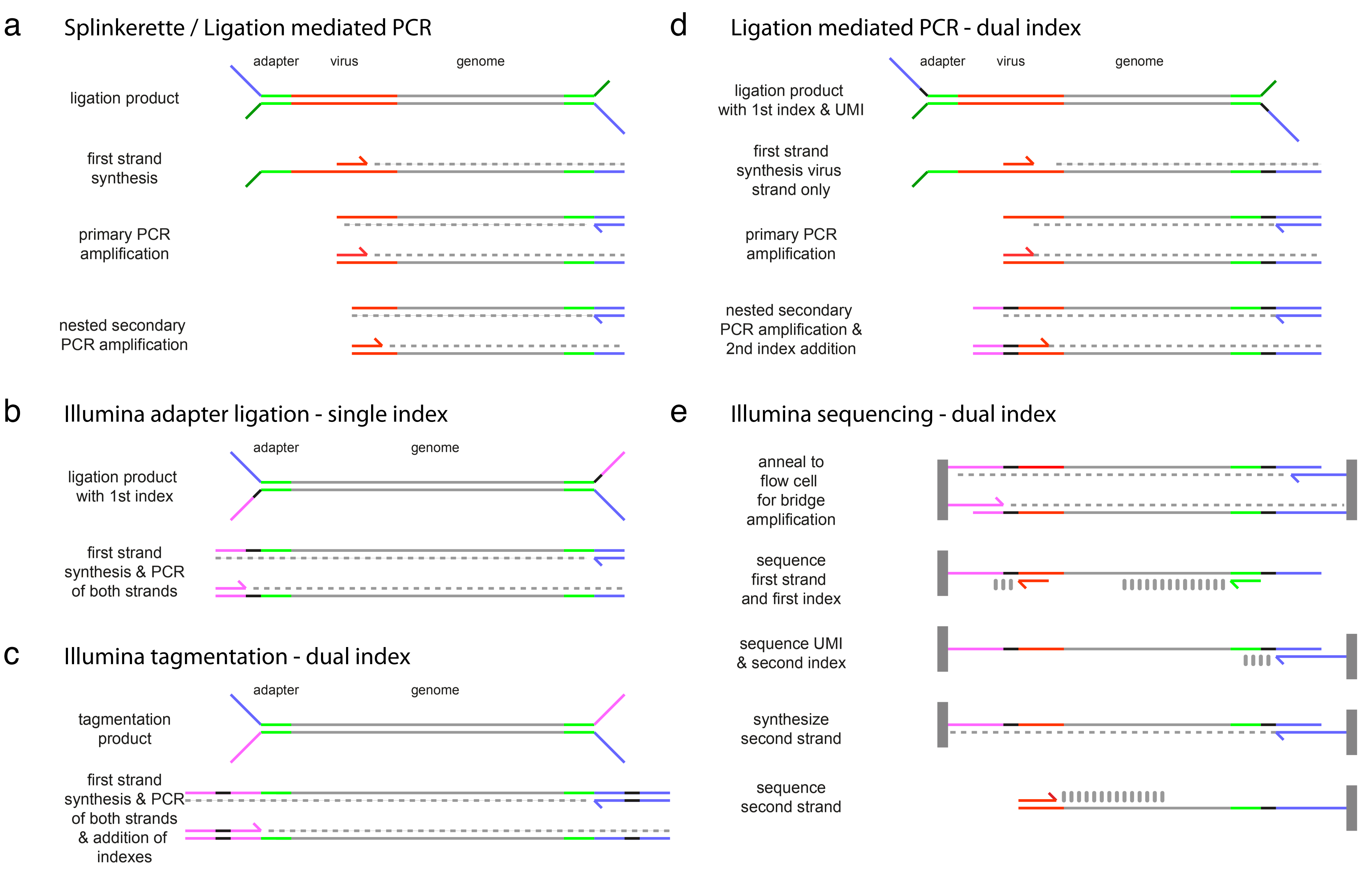
a
Splinkerette / Ligation mediated PCR
adapter
virus
genome
ligation product
first strand synthesis
primary PCR amplification
nested secondary PCR amplification
b
Illumina adapter ligation - single index
adapter
genome
ligation product with 1st index
first strand synthesis & PCR of both strands
c
Illumina tagmentation - dual index
adapter
genome
tagmentation product
first strand synthesis & PCR of both strands & addition of indexes
d
Ligation mediated PCR - dual index
adapter
virus
genome
ligation product with 1st index & UMI
first strand synthesis virus strand only
primary PCR amplification
nested secondary PCR amplification & 2nd index addition
e
Illumina sequencing - dual index
anneal to flow cell for bridge amplification
sequence first strand and first index
sequence UMI & second index
synthesize second strand
sequence second strand

Supplementary Figure 3 - Comparison of a novel protocol for cloning and next generation sequencing of retroviral integration sites with previous methods.

a) The steps of traditional ligation mediated PCR strategy using adapters with non-complementary segments and two rounds of nested PCR. The adapter strands are partially non-complementary and the lower strand (dark green) has no complementary primer. The adapter primer (blue) cannot bind to a template until the first strand has been synthesized from the virus primer (red). Subsequent steps will amplify virus flanked genomic regions but not other regions. b) Standard Illumina library preparation protocols for single index libraries. Using ligation of adapters, an index is included in the adapter for each library, with one copy per fragment being present in the final product. Both strands are amplified simultaneously. c) Illumina Nextera library prep using tagmentation. Adapters are added via T5 transposase. Both strands are amplified simultaneously using primer pairs that add an index at each end. d) Hybrid protocol for ligation mediated PCR incorporating one index in the adapter and one in the secondary PCR step. The placement of the index is switched from the strand normally used in Illumina adapters such that it will be retained after the first strand synthesis from the virus primer. e) A modified dual index Nextera sequencing protocol is used with custom primers and modified numbers of bases read from each index depending on the length of the custom index (our protocol uses 10bp length indexes). The custom virus primer can be nested back from the virus genome junction to allow the junction to be sequenced. By making the virus genome junction the second read, the uniformity of the virus bases does not cause loss of cluster fidelity.

a

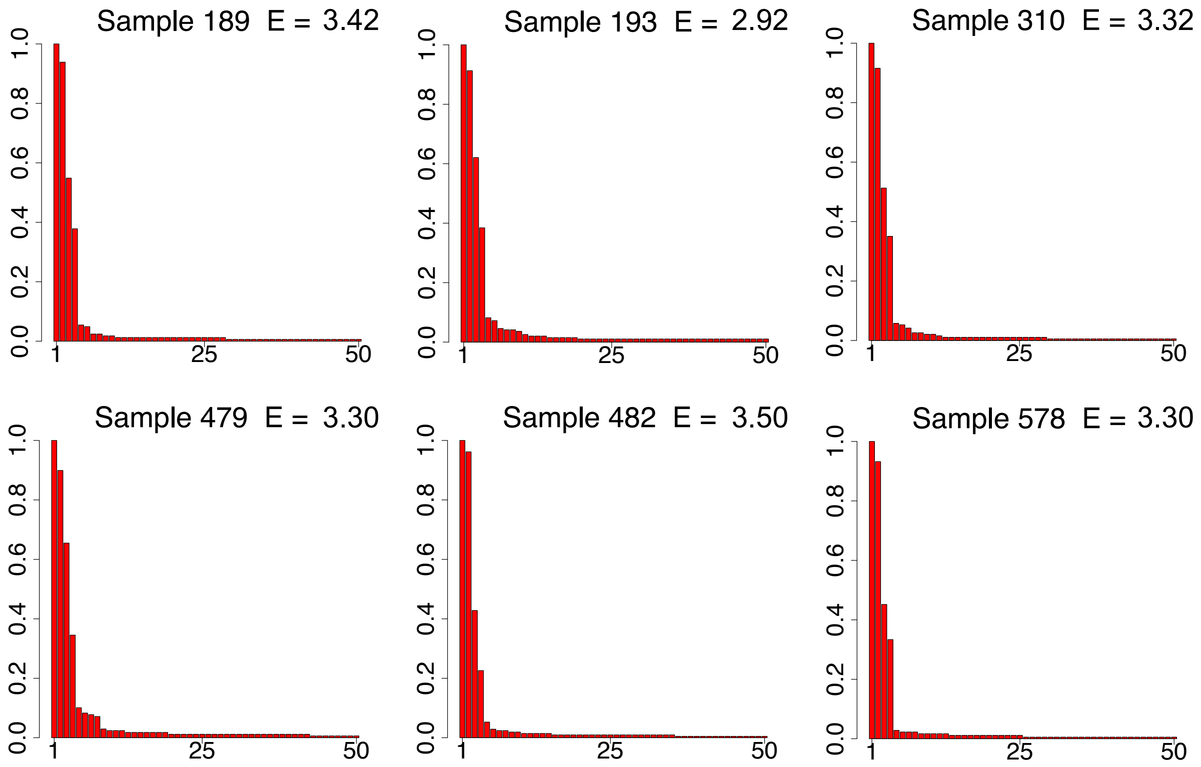

b

	library #189	library #193	library #310	library #479	library #482	library #578
total reads	21281	70931	26838	35199	27798	7254
total fragments	1017	1464	1080	1153	997	876
total inserts	508	789	494	568	398	364
singletons	480	739	465	526	363	339
non singletons	28	50	29	42	35	25
present in 1 library	482	765	468	535	371	337
present in >1 library	26	24	26	33	27	27
present in 2 libraries	10	10	8	16	13	11
present in 3 libraries	3	2	6	5	2	3
present in 4 libraries	1	0	1	0	1	1
present in 5 libraries	2	2	1	2	1	2
present in 6 libraries	10	10	10	10	10	10

c

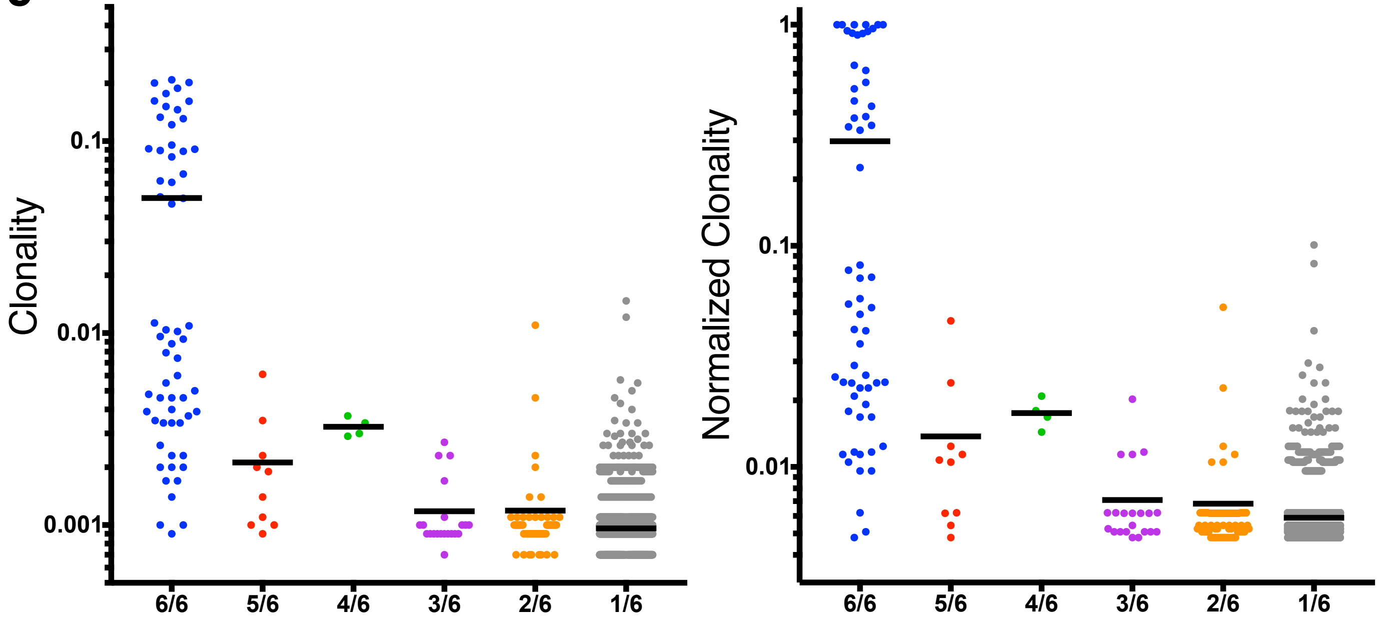

Supplementary Figure 4 – Replicate libraries from mouse 3194 spleen DNA.

a) Clonality profile shape is consistent between six replicate libraries of a DNA sample containing four clonal integrations. The average entropy score for these profiles (E) is 3.29. b) Summary statistics of each library and the overlap of insertions between libraries. Although the vast majority of sub clonal mutations were found in only one library, 20 of 21 insertions above NC 0.1 were found present in all six libraries. Only 1 of 2958 insertions found in only one library had NC <0.1. c) The set of mutations present in only one of the six libraries had a much lower average clonality/normalized clonality value (horizontal line) than those inserts found in more than one library. Many low clonality insertions are also found in more than one library.

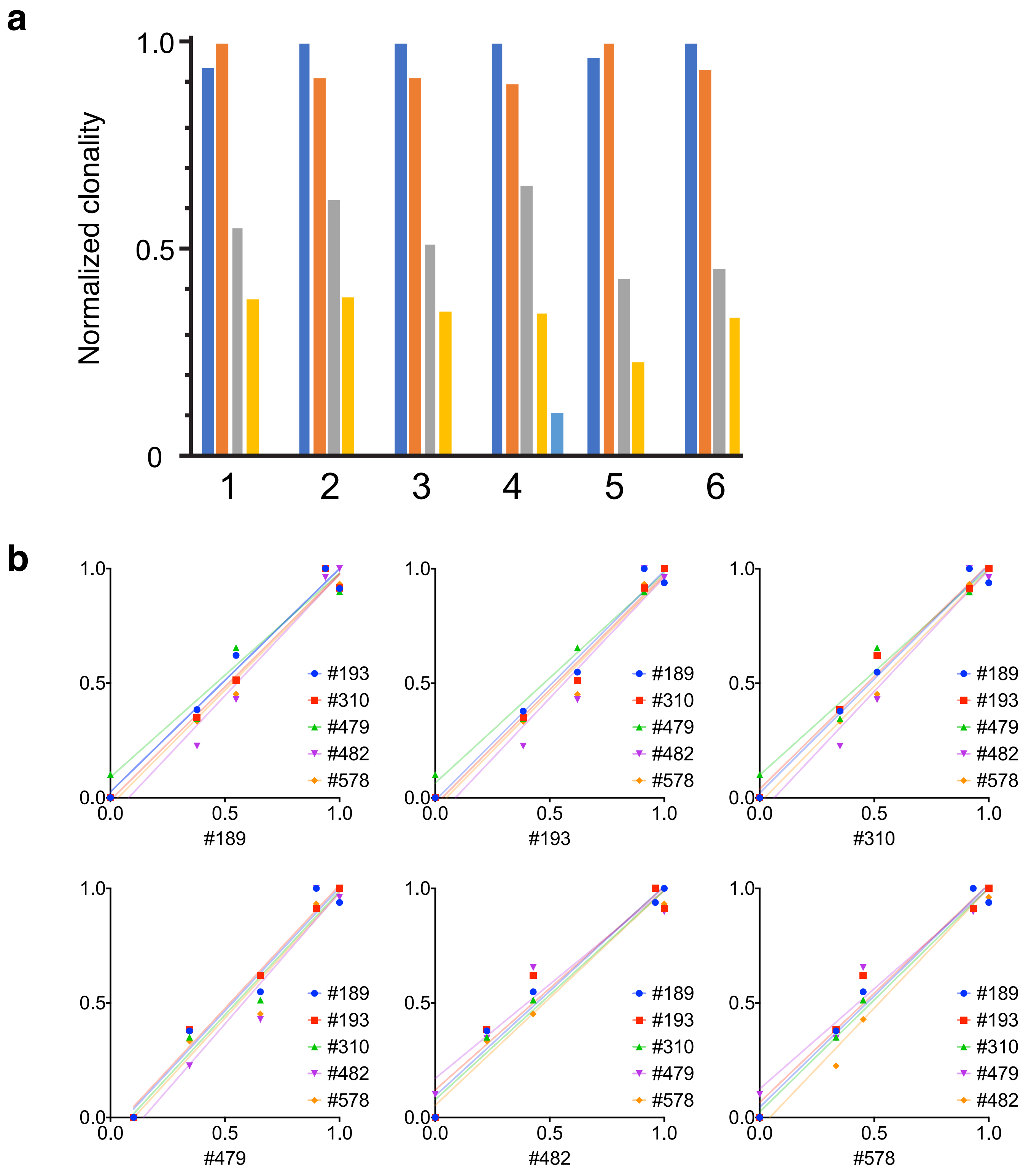

c

	#189	#193	#310	#479	#482	#578
**#189**	-	0.9	0.9	0.9	1	0.9
**#193**	0.9	-	1	1	0.9	1
**#310**	0.9	1	-	1	0.9	1
**#479**	0.9	1	1	-	0.9	1
**#482**	1	0.9	0.9	0.9	-	0.9
**#578**	0.9	1	1	1	0.9	-

Pairwise Spearman correlation values

Supplementary Figure 5 – Replicate libraries from mouse 3194 spleen DNA.

a) Graph of the matched normalized clonality values for the 21 inserts with NC <0.1 at 5 locations. b) The normalized clonality values for each library are plotted against the other 5 libraries. c) Spearman correlation coefficients for pairwise comparison between all samples using NC values for all inserts with NC > 0.1. Samples lacking the 5th insert are assigned a value of zero for that insert.

a

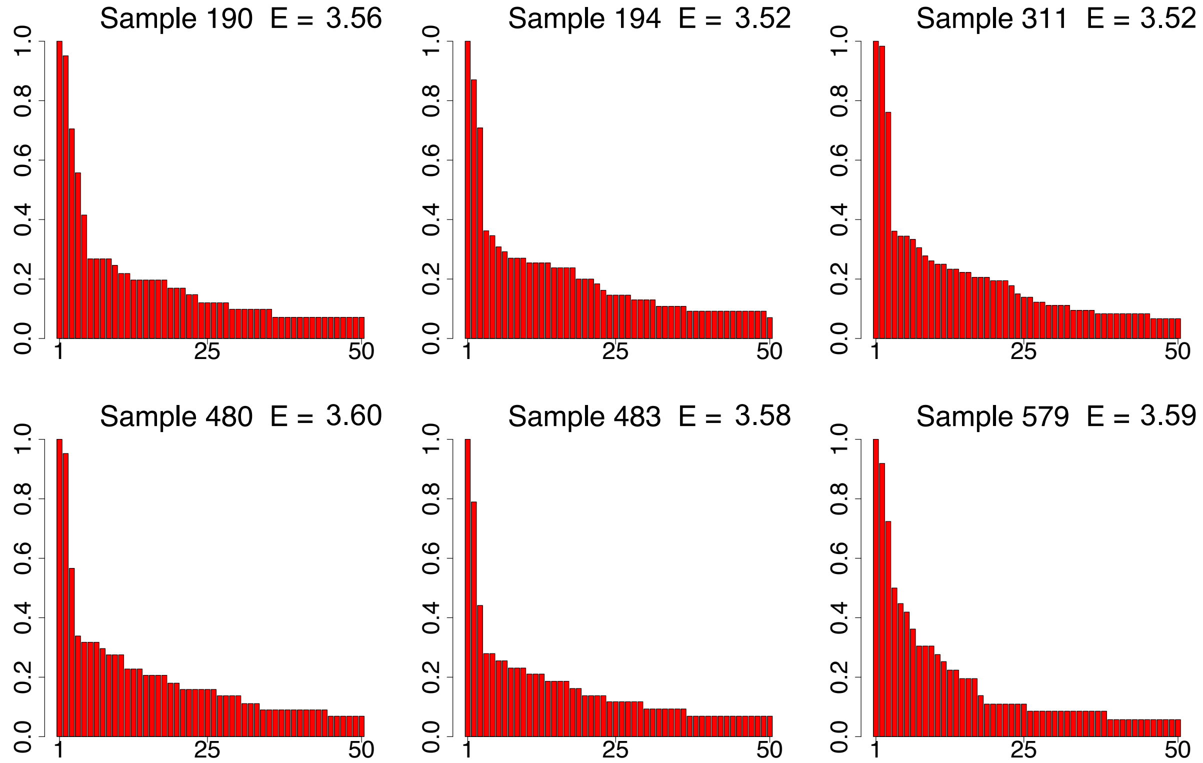

b

	library #190	library #194	library #311	library #480	library #483	library #579
total reads	37412	69541	50164	61953	26292	8675
total fragments	2244	2975	4007	2330	1742	1711
total inserts	1791	2199	3035	1793	1336	1372
singletons	1645	1972	2789	1626	1202	1275
non singletons	146	227	246	167	134	97
present in 1 library	1566	1934	2703	1558	1143	1162
present in >1 library	225	265	332	235	193	210
present in 2 libraries	89	115	167	106	70	93
present in 3 libraries	35	50	56	33	30	30
present in 4 libraries	32	33	39	31	26	23
present in 5 libraries	27	25	28	23	25	22
present in 6 libraries	42	42	42	42	42	42

c

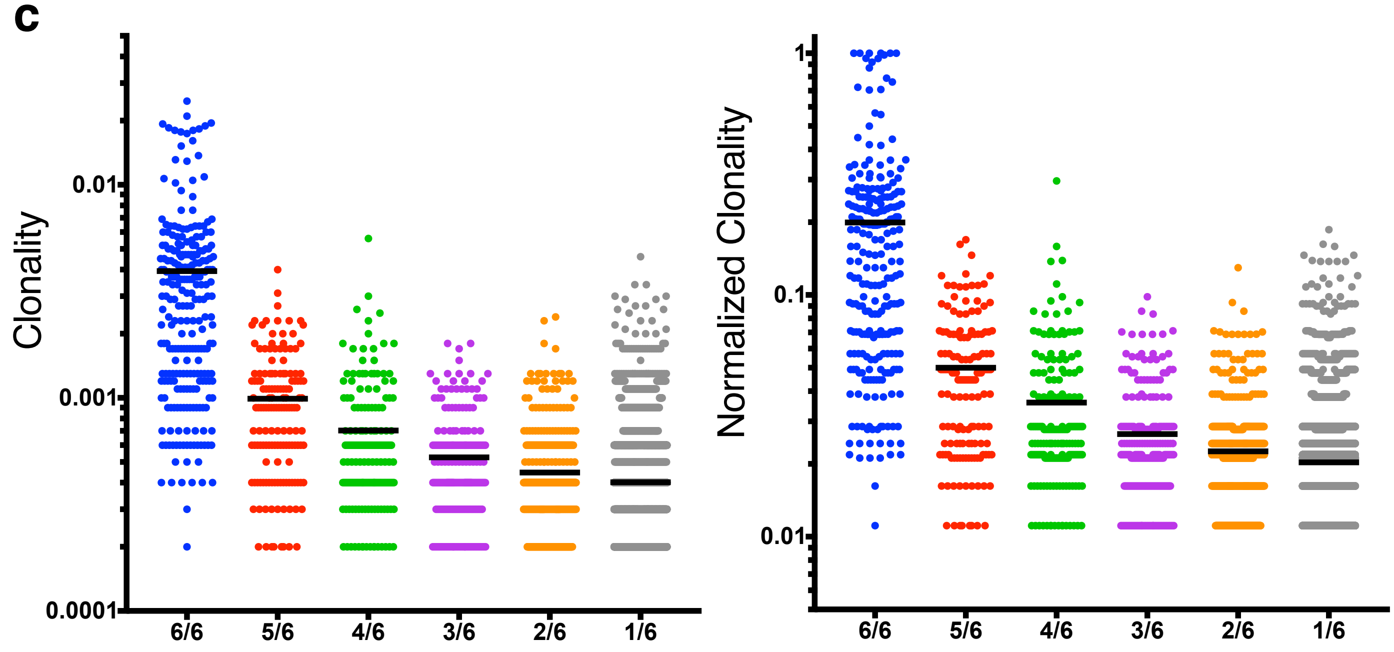

Supplementary Figure 6 – Replicate libraries from mouse 3195 spleen DNA.

a) Clonality profile shape is consistent between six replicate libraries of a DNA sample containing between 25 and 30 clonal integrations. The average entropy score for these profiles (E) is 3.56. b) Summary statistics of each library and the overlap of insertions between libraries. Although the vast majority of sub clonal mutations were found in only one library, 143 of 181 insertions above NC 0.1 were found present in all six libraries. Only 18 of 10066 insertions found in only one library had NC <0.1. c) The set of mutations present in only one of the six libraries had a much lower average clonality/normalized clonality value (horizontal line) than those inserts found in more than one library. Many low clonality insertions are also found in more than one library.

a

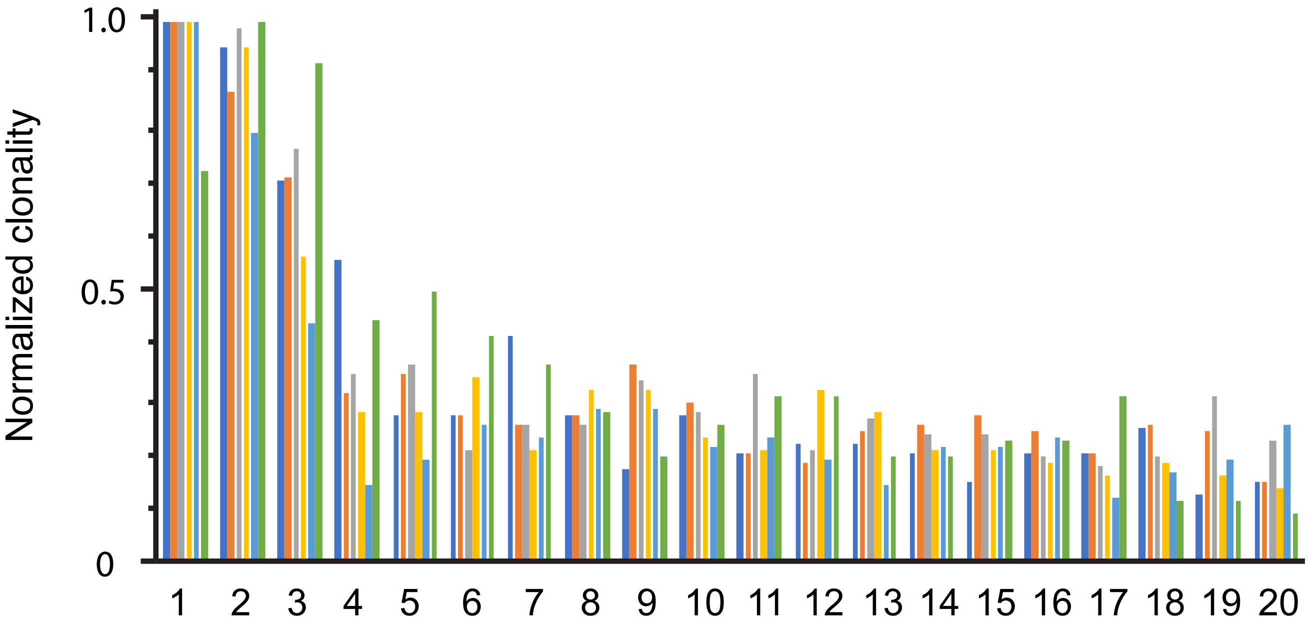

b

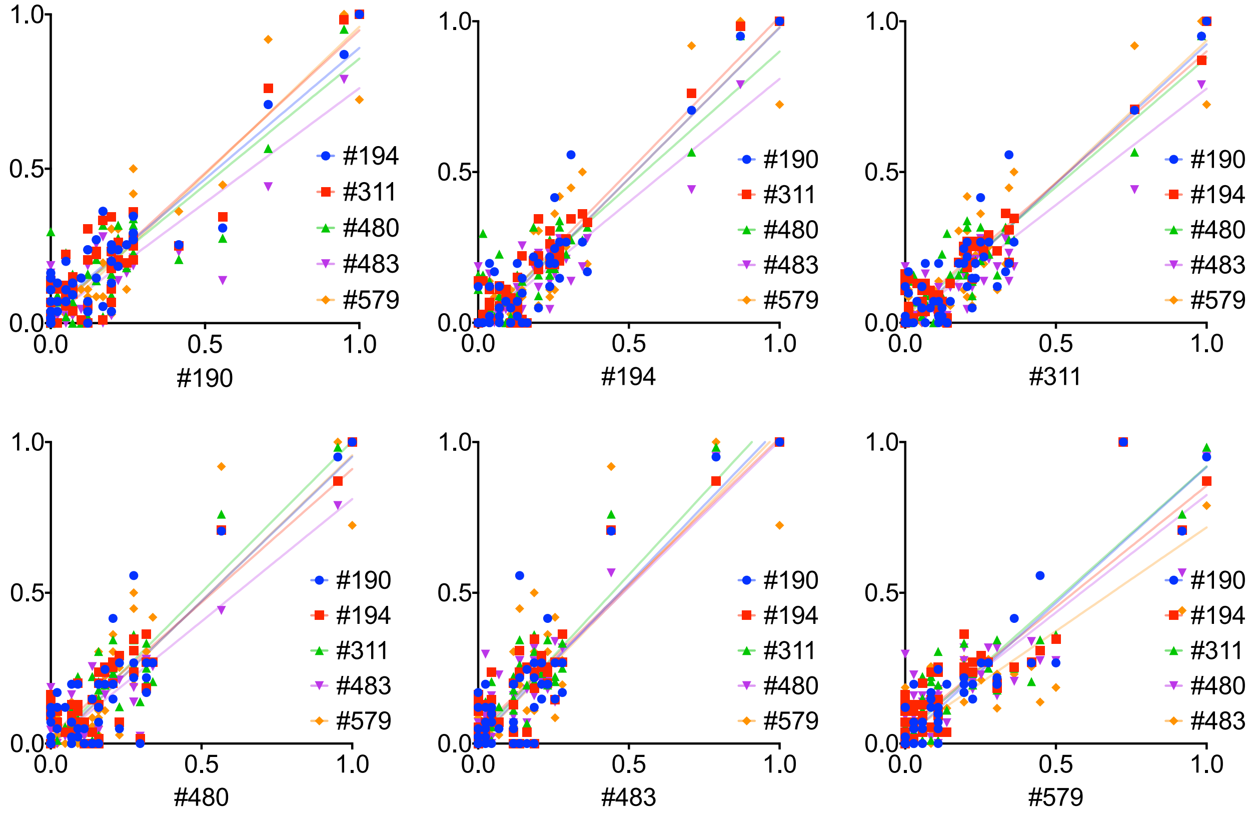

c

	#190	#194	#311	#480	#483	#579
**#190**		0.78	0.81	0.67	0.7	0.85
**#194**	0.78		0.77	0.61	0.64	0.79
**#311**	0.81	0.77		0.8	0.76	0.85
**#480**	0.67	0.61	0.8		0.62	0.73
**#483**	0.7	0.64	0.76	0.62		0.77
**#579**	0.85	0.79	0.85	0.73	0.77	

Pairwise Spearman correlation values

Supplementary Figure 7 – Replicate libraries from mouse 3195 spleen DNA.

a) Graph of the matched normalized clonality values for the 120 inserts with NC <0.1 at the 20 most clonal locations (measured by average normalized clonality) all of which were measured found in all 6 libraries. b) The normalized clonality values for each library are plotted against the other 5 libraries. c) Spearman correlation coefficients for pairwise comparison between all samples using NC values for all inserts with NC > 0.1. Samples lacking any of the inserts are assigned a value of zero for that insert.

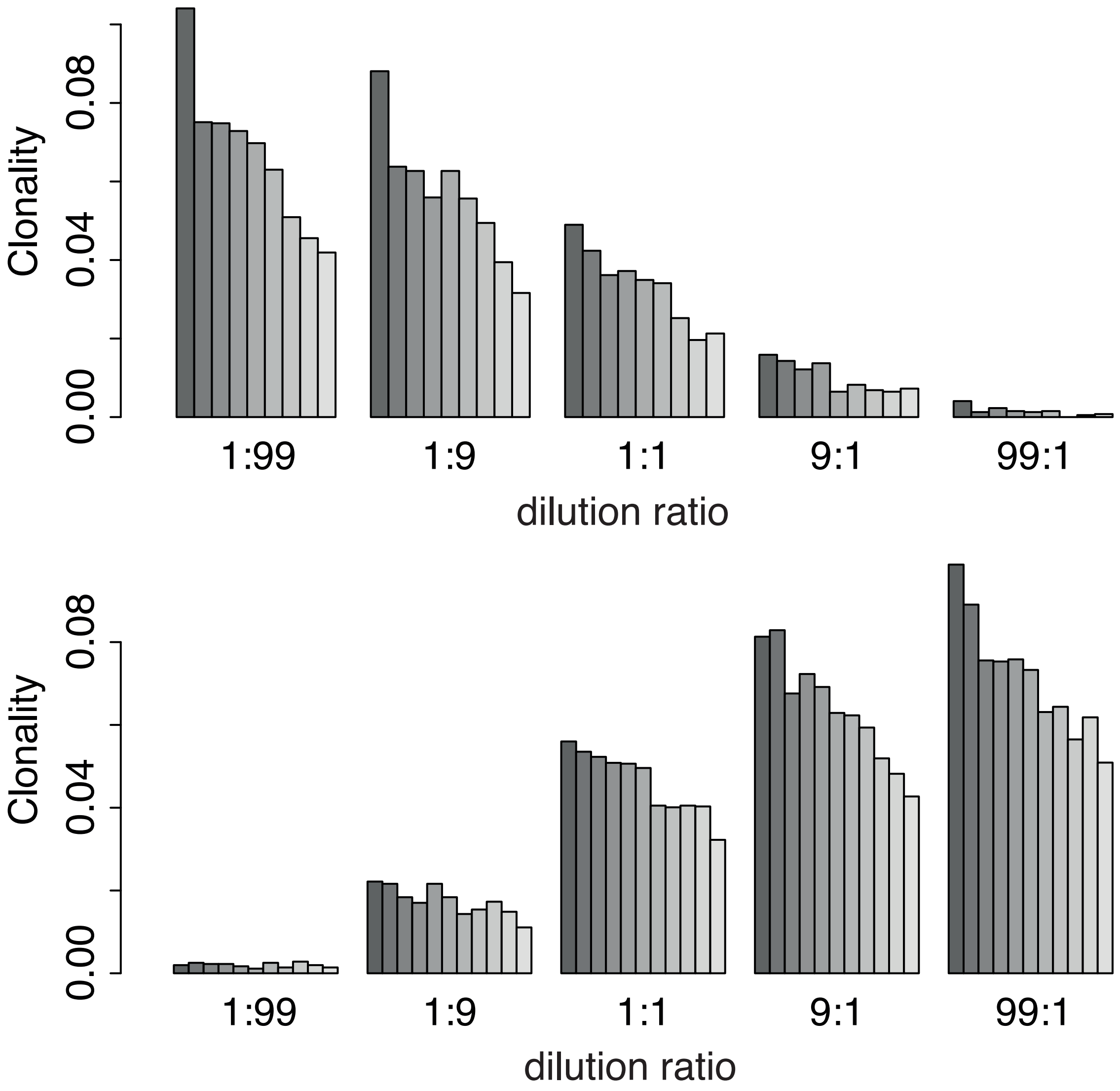
Clonality
0.00
0.04
0.08
1:99
1:9
1:1
9:1
99:1
dilution ratio
Clonality
0.00
0.04
0.08
1:99
1:9
1:1
9:1
99:1
dilution ratio

Supplementary Figure 8 – Insert cloning in reciprocally diluted DNA samples.

A dilution series was prepared of two lymphoma DNAs into each other at different ratios (1 in 100, 1 in 10 and 1:1). Grey bars indicate the clonality values of the most abundant integrations in the two different DNA samples. When mixed together in different ratios the set of integrations from each sample becomes diluted but remains detectable even at dilution factor of 1 in 100.

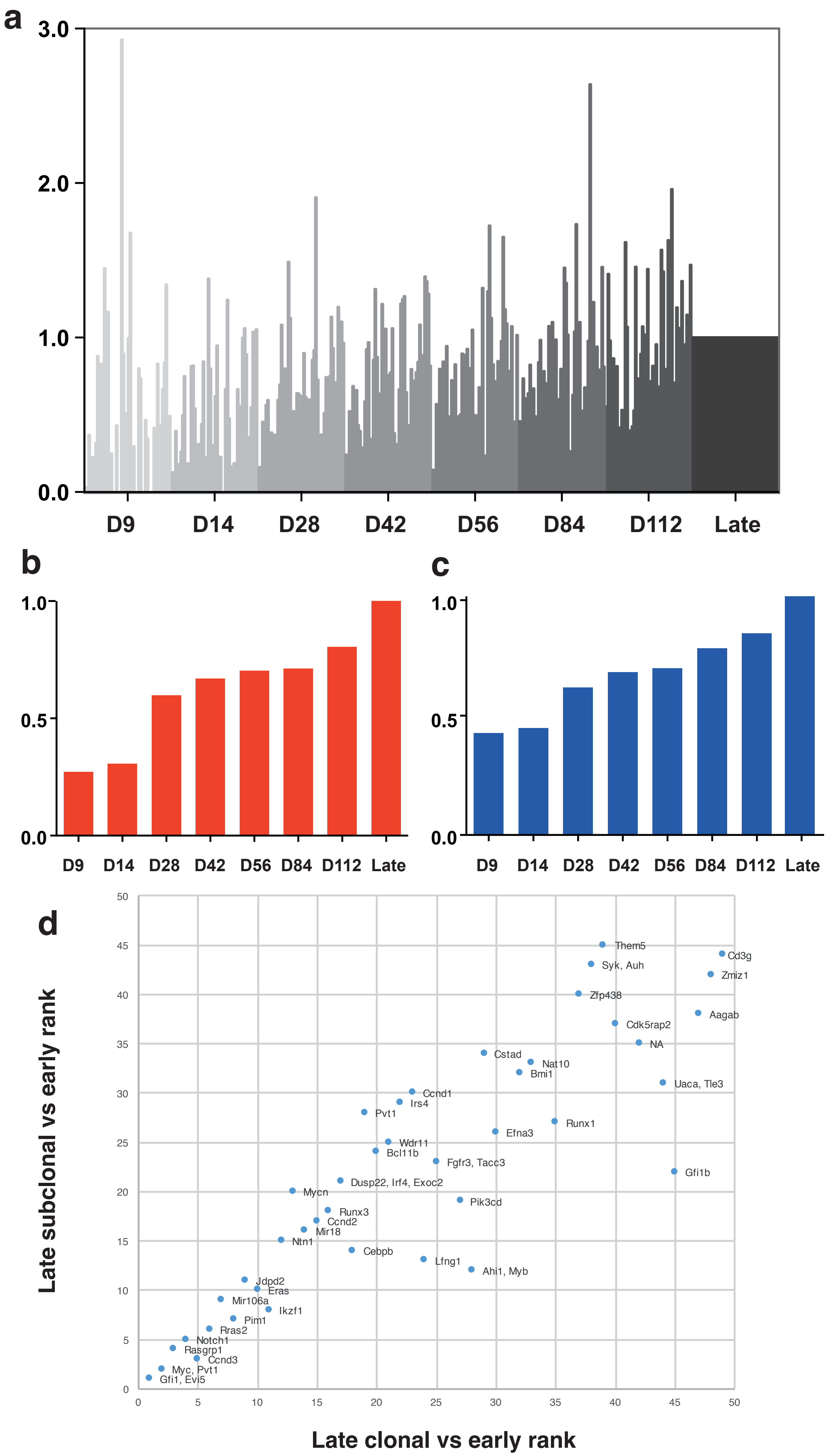
a
3.0
2.0
1.0
0.0
D9
D14
D28
D42
D56
D84
D112
Late
b
1.0
0.5
0.0
D9
D14
D28
D42
D56
D84
D112
Late
c
1.0
0.5
0.0
D9
D14
D28
D42
D56
D84
D112
Late
d
Late subclonal vs early rank
Late clonal vs early rank
Them5
Cd3g
Syk, Auh
Zmiz1
Zfp438
Aagab
Cdk5rap2
NA
Cstad
Nat10
Bmi1
Uaca, Tle3
Ccnd1
Irs4
Pvt1
Runx1
Efna3
Wdr11
Bcl11b
Fgfr3, Tacc3
Gfi1b
Dusp22, Irf4, Exoc2
Mycn
Pik3cd
Runx3
Ccnd2
Mir18
Ntn1
Cebpb
Lfng1
Ahi1, Myb
Jdpd2
Eras
Mir106a
Ikzf1
Pim1
Rras2
Notch1
Rasgrp1
Ccnd3
Myc, Pvt1
Gfi1, Evi5

Supplementary Figure 9 – Selection of integrations at CIS loci over a time course is observed in both clonal and subclonal populations.

a) For each of the top 50 clonal insert GKC CIS loci the relative abundance of all integrations at each locus is plotted at each time point over the time course. For each data point we calculate the fraction of the total inserts at that locus in all samples at that time point. Then for each locus all datapoints at different time points are normalized to give a value of 1 in late stage lymphomas. Some loci are more frequently mutated in time points than in the late stage lymphoma samples however lower panels of the average (b) and median (c) values for the top 50 loci combined indicate a consistent increase in mutation frequency for the aggregate data. d) When loci are ranked by the ratio of late mutations to early mutations (assigning a p-value using Fisher's exact tests to determine the rank) this yields a near identical order whether using late stage clonal integrations or late stage subclonal integrations. The correlation becomes less pronounced for lower ranked loci.

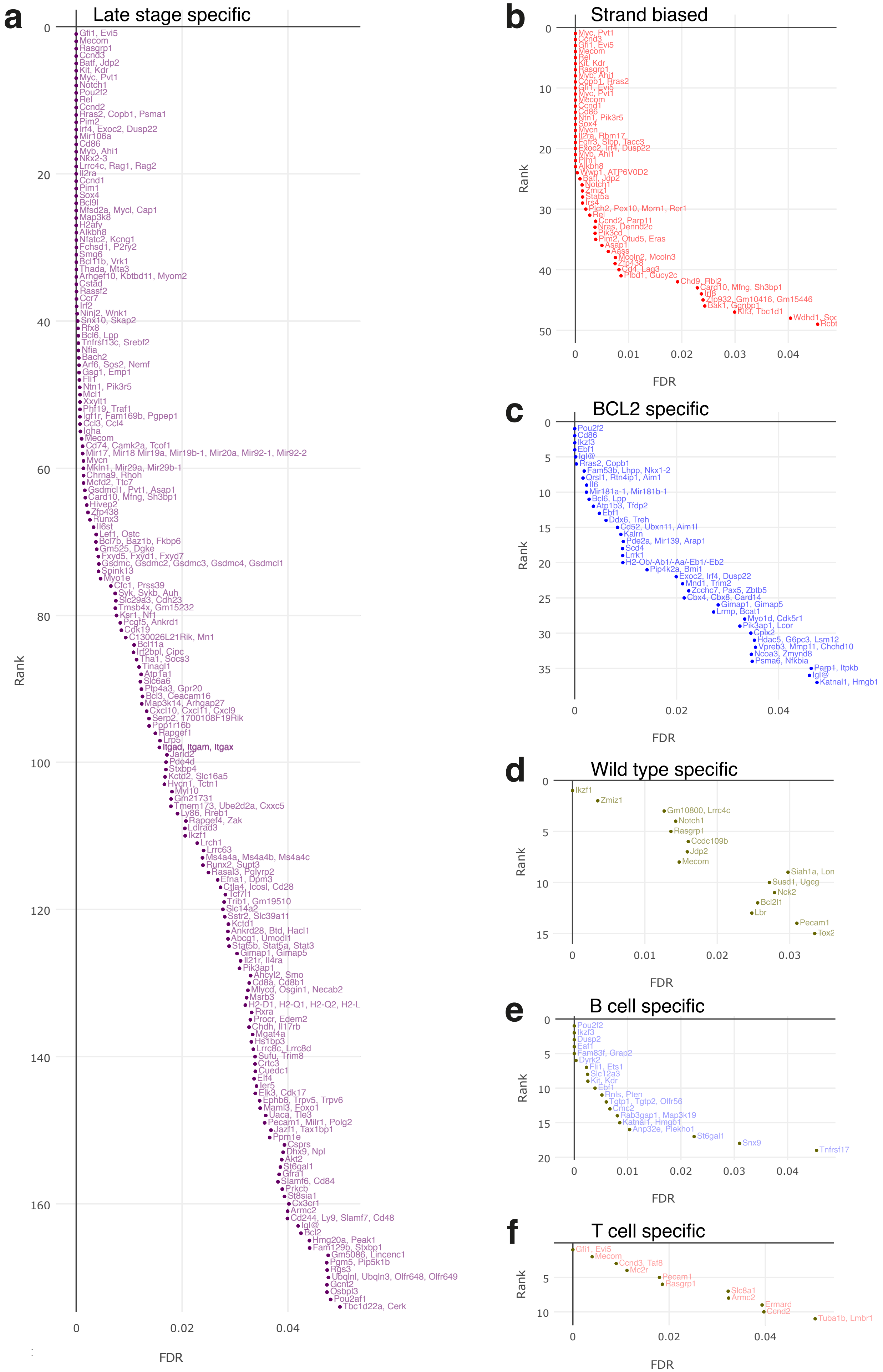
a
Late stage specific
Rank
FDR
0
20
40
60
80
100
120
140
160
0
0.02
0.04
Gfi1, Evi5
Mecom
Rasgrp1
Ccnd3
Batf, Jdp2
Kit, Kdr
Myc, Pvt1
Notch1
Pou2f2
Rel
Ccnd2
Rras2, Copb1, Psma1
Pim2
Irf4, Exoc2, Dusp22
Mir106a
Cd86
Myb, Ahi1
Nkx2-3
Lrrc4c, Rag1, Rag2
Il2ra
Ccnd1
Pim1
Sox4
Bcl9l
Mfsd2a, Mycl, Cap1
Map3k8
H2afy
Alkbh8
Nfatc2, Kcng1
Fchsd1, P2ry2
Smg6
Bcl11b, Vrk1
Thada, Mta3
Arhgef10, Kbtbd11, Myom2
Cstad
Rassf2
Ccr7
Irf2
Ninj2, Wnk1
Snx10, Skap2
Rfx8
Bcl6, Lpp
Tnfrsf13c, Srebf2
Nfia
Bach2
Arf6, Sos2, Nemf
Gsg1, Emp1
Fli1
Ntn1, Pik3r5
Mcl1
Xxylt1
Phf19, Traf1
Igf1r, Fam169b, Pgpep1
Ccl3, Ccl4
Igha
Mecom
Cd74, Camk2a, Tcof1
Mir17, Mir18 Mir19a, Mir19b-1, Mir20a, Mir92-1, Mir92-2
Mycn
Mkln1, Mir29a, Mir29b-1
Chrna9, Rhoh
Mcfd2, Ttc7
Gsdmcl1, Pvt1, Asap1
Card10, Mfng, Sh3bp1
Hivep2
Zfp438
Runx3
Il6st
Lef1, Ostc
Bcl7b, Baz1b, Fkbp6
Gm525, Dgke
Fxyd5, Fxyd1, Fxyd7
Gsdmc, Gsdmc2, Gsdmc3, Gsdmc4, Gsdmcl1
Spink13
Myo1e
Cfc1, Prss39
Syk, Sykb, Auh
Slc29a3, Cdh23
Tmsb4x, Gm15232
Ksr1, Nf1
Pcgf5, Ankrd1
Cdk19
C130026L21Rik, Mn1
Bcl11a
Irf2bpl, Cipc
Tha1, Socs3
Tinagl1
Atp1a1
Slc6a6
Ptp4a3, Gpr20
Bcl3, Ceacam16
Map3k14, Arhgap27
Cxcl10, Cxcl11, Cxcl9
Serp2, 1700108F19Rik
Ppp1r16b
Rapgef1
Lrp5
Itgad, Itgam, Itgax
Jarid2
Pde4d
Stxbp4
Kctd2, Slc16a5
Hvcn1, Tctn1
Myl10
Gm21731
Tmem173, Ube2d2a, Cxxc5
Ly86, Rreb1
Rapgef4, Zak
Ldlrad3
Ikzf1
Lrch1
Lrrc63
Ms4a4a, Ms4a4b, Ms4a4c
Runx2, Supt3
Rasal3, Pglyrp2
Efna1, Dpm3
Ctla4, Icosl, Cd28
Tcf7l1
Trib1, Gm19510
Slc14a2
Sstr2, Slc39a11
Kctd1
Ankrd28, Btd, Hacl1
Abcg1, Umodl1
Stat5b, Stat5a, Stat3
Gimap1, Gimap5
Il21r, Il4ra
Pik3ap1
Ahcyl2, Smo
Cd8a, Cd8b1
Mlycd, Osgin1, Necab2
Msrb3
H2-D1, H2-Q1, H2-Q2, H2-L
Rxra
Procr, Edem2
Chdh, Il17rb
Mgat4a
Hs1bp3
Lrrc8c, Lrrc8d
Sufu, Trim8
Crtc3
Cuedc1
Elf4
Ier5
Elk3, Cdk17
Ephb6, Trpv5, Trpv6
Maml3, Foxo1
Uaca, Tle3
Pecam1, Milr1, Polg2
Jazf1, Tax1bp1
Ppm1e
Csprs
Dhx9, Npl
Akt2
St6gal1
Gfra1
Slamf6, Cd84
Prkcb
St8sia1
Cx3cr1
Armc2
Cd244, Ly9, Slamf7, Cd48
Igl@
Bcl2
Hmg20a, Peak1
Fam129b, Stxbp1
Gm5086, Lincenc1
Pgm5, Pip5k1b
Rgs3
Ubqlnl, Ubqln3, Olfr648, Olfr649
Gcnt2
Osbpl3
Pou2af1
Tbc1d22a, Cerk
b
Strand biased
Rank
FDR
0
10
20
30
40
50
0
0.01
0.02
0.03
0.04
Myc, Pvt1
Ccnd3
Gfi1, Evi5
Mecom
Rel
Kit, Kdr
Rasgrp1
Myb, Ahi1
Copb1, Rras2
Gfi1, Evi5
Myc, Pvt1
Mecom
Ccnd1
Cd86
Ntn1, Pik3r5
Sox4
Mycn
Il2ra, Rbm17
Egfr3, Slbp, Tacc3
Exoc2, Irf4, Dusp22
Myb, Ahi1
Pim1
Alkbh8
Wwp1, ATP6V0D2
Batf, Jdp2
Notch1
Zmiz1
Stat5a
Irs4
Plch2, Pex10, Morn1, Rer1
Rel
Ccnd2, Parp11
Nras, Dennd2c
Pik3cd
Pim2, Otud5, Eras
Asap1
Adss
Mcoln2, Mcoln3
Zfp438
Cd4, Lag3
Plbd1, Gucy2c
Chd9, Rbl2
Card10, Mfng, Sh3bp1
Irf8
Zfp932, Gm10416, Gm15446
Bak1, Ggnbp1
Klf3, Tbc1d1
Wdhd1, Soc
Rcbl
c
BCL2 specific
Rank
FDR
0
5
10
15
20
25
30
35
0
0.02
0.04
Pou2f2
Cd86
Ikzf3
Ebf1
Igl@
Rras2, Copb1
Fam53b, Lhpp, Nkx1-2
Orsl1, Rtn4ip1, Aim1
Il6
Mir181a-1, Mir181b-1
Bcl6, Lpp
Atp1b3, Tfdp2
Ebf1
Ddx6, Treh
Cd52, Ubxn11, Aim1l
Kalrn
Pde2a, Mir139, Arap1
Scd4
Lrrk1
H2-Ob/-Ab1/-Aa/-Eb1/-Eb2
Pip4k2a, Bmi1
Exoc2, Irf4, Dusp22
Mnd1, Trim2
Zcchc7, Pax5, Zbtb5
Cbx4, Cbx8, Card14
Gimap1, Gimap5
Lrmp, Bcat1
Myo1d, Cdk5r1
Pik3ap1, Lcor
Cplx2
Hdac5, G6pc3, Lsm12
Vpreb3, Mmp11, Chchd10
Ncoa3, Zmynd8
Psma6, Nfkbia
Parp1, Itpkb
Igl@
Katnal1, Hmgb1
d
Wild type specific
Rank
FDR
0
5
10
15
0
0.01
0.02
0.03
Ikzf1
Zmiz1
Gm10800, Lrrc4c
Notch1
Rasgrp1
Ccdc109b
Jdp2
Mecom
Siah1a, Lon
Susd1, Ugcg
Nck2
Bcl2l1
Lbr
Pecam1
Tox2
e
B cell specific
Rank
FDR
0
5
10
15
20
0
0.01
0.02
0.03
0.04
Pou2f2
Ikzf3
Dusp2
Eaf1
Fam83f, Grap2
Dyrk2
Fli1, Ets1
Slc12a3
Kit, Kdr
Ebf1
Rnls, Pten
Tgtp1, Tgtp2, Olfr56
Cmc2
Rab3gap1, Map3k19
Katnal1, Hmgb1
Anp32e, Plekho1
St6gal1
Snx9
Tnfrsf17
f
T cell specific
Rank
FDR
5
10
0
0.02
0.04
Gfi1, Evi5
Mecom
Ccnd3, Taf8
Mc2r
Pecam1
Rasgrp1
Slc8a1
Armc2
Ermard
Ccnd2
Tuba1b, Lmbr1

Supplementary Figure 10 – All loci selected by different criteria

Plots of all candidate loci identified through genome wide scans for selection. Each locus is ranked by p-value and plotted against their false discovery rate. All loci with a false discovery rate of less than 0.05 are listed. a) Early vs Late selection. b) Strand biased loci. c) Bcl2 specific loci. d) Wild type specific loci. e) B cell specific loci. f) T cell specific loci. Many loci are implicated by more than one criteria as indicated in Figure 5a.

a

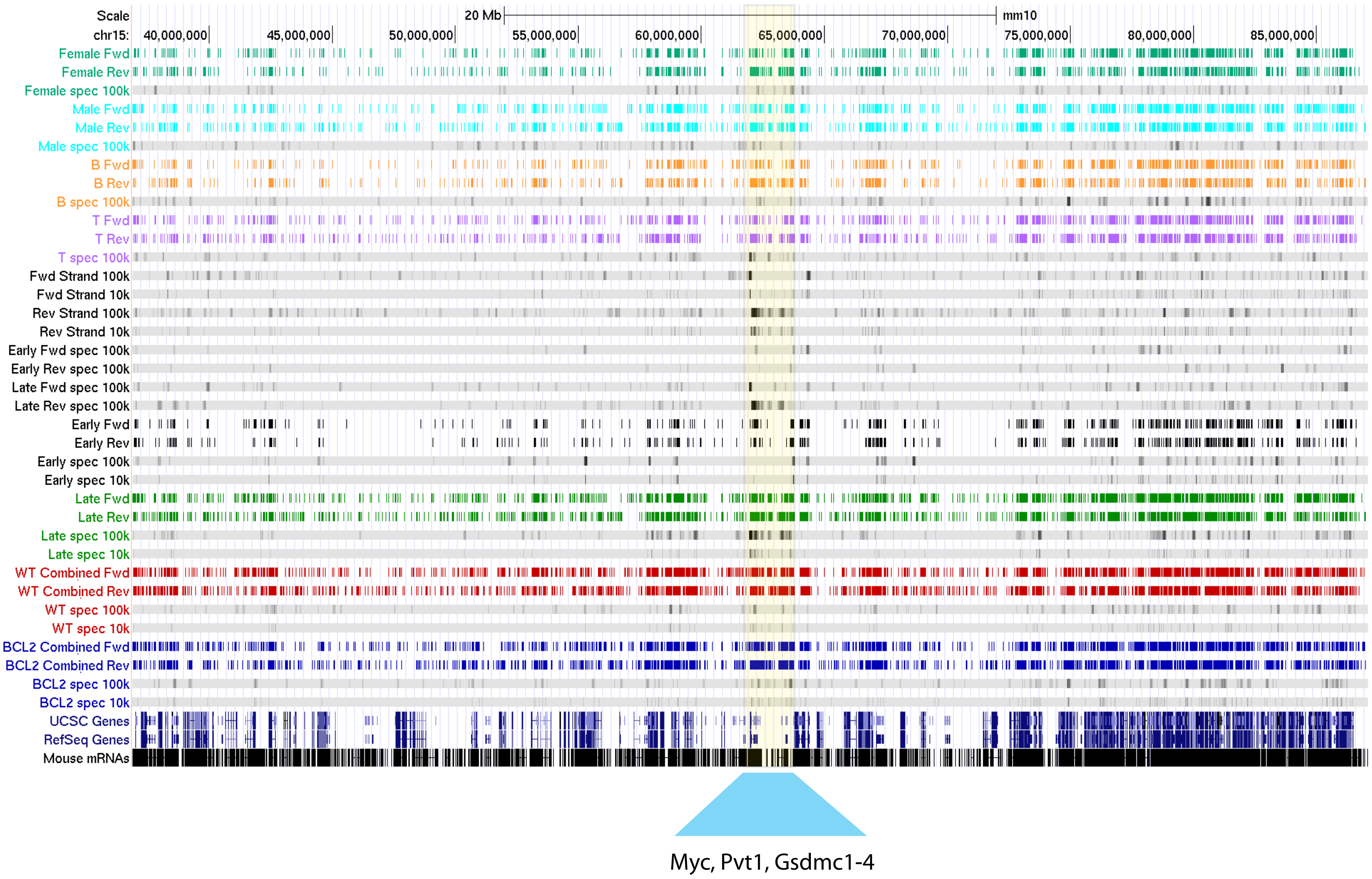

Scale
chr15:
20 Mb
mm10
40,000,000
45,000,000
50,000,000
55,000,000
60,000,000
65,000,000
70,000,000
75,000,000
80,000,000
85,000,000
Female Fwd
Female Rev
Female spec 100k
Male Fwd
Male Rev
Male spec 100k
B Fwd
B Rev
B spec 100k
T Fwd
T Rev
T spec 100k
Fwd Strand 100k
Fwd Strand 10k
Rev Strand 100k
Rev Strand 10k
Early Fwd spec 100k
Early Rev spec 100k
Late Fwd spec 100k
Late Rev spec 100k
Early Fwd
Early Rev
Early spec 100k
Early spec 10k
Late Fwd
Late Rev
Late spec 100k
Late spec 10k
WT Combined Fwd
WT Combined Rev
WT spec 100k
WT spec 10k
BCL2 Combined Fwd
BCL2 Combined Rev
BCL2 spec 100k
BCL2 spec 10k
UCSC Genes
RefSeq Genes
Mouse mRNAs
Myc, Pvt1, Gsdmc1-4

b chr15:61,930,000-63,930,000 - Myc, Pvt1, Gsdmc gene cluster

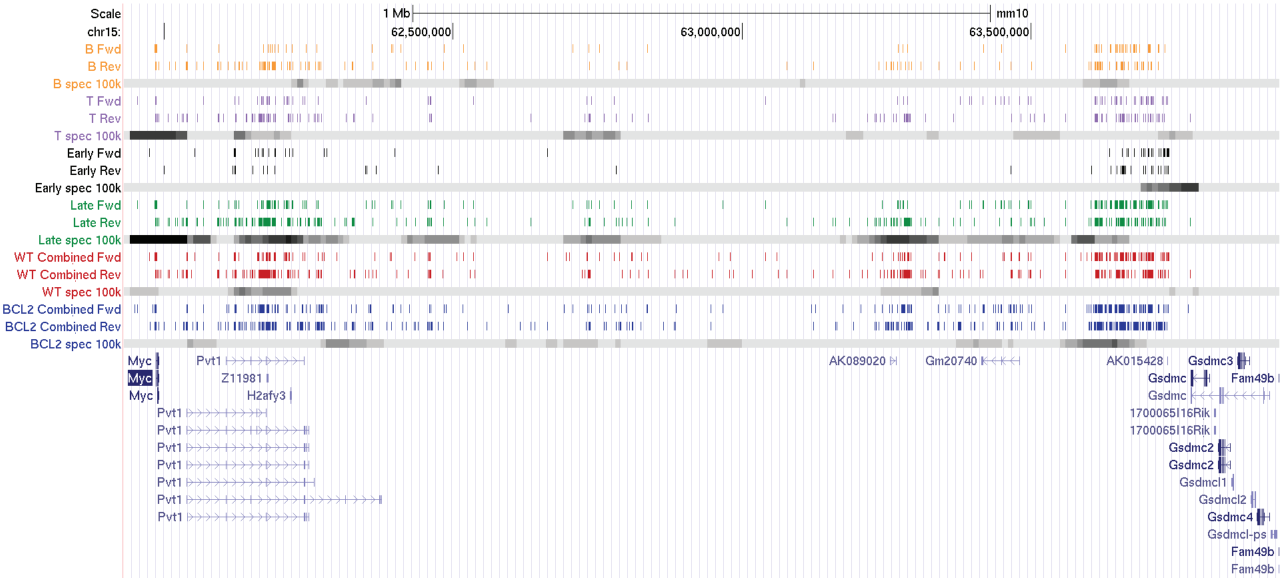

c chr11:23,650,000-24,300,000 Rel, Papolg, Bcl11a

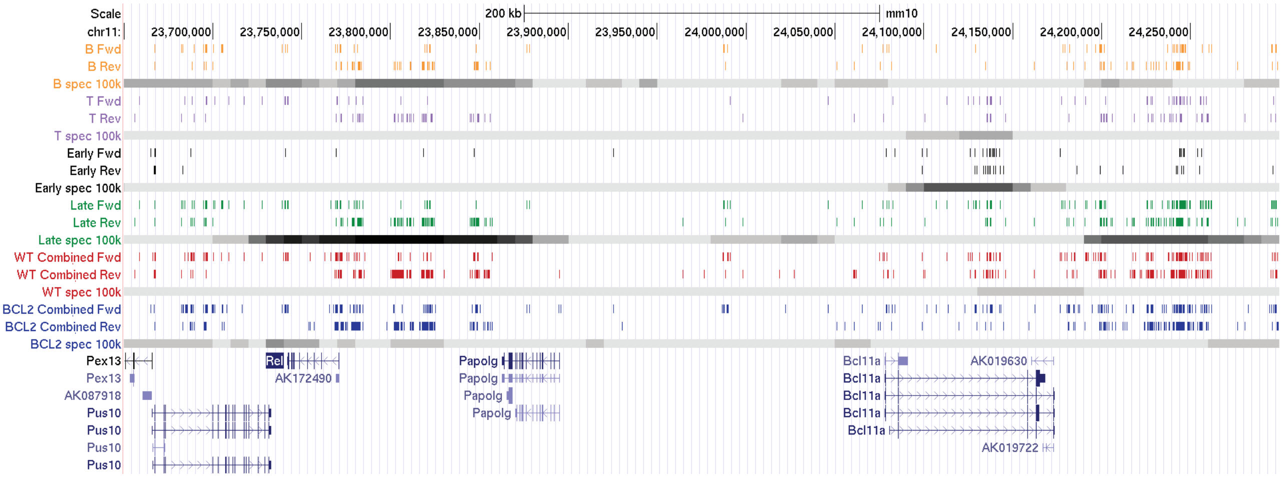

d chr1:171,500,000-172,000,000 - Slamf gene cluster

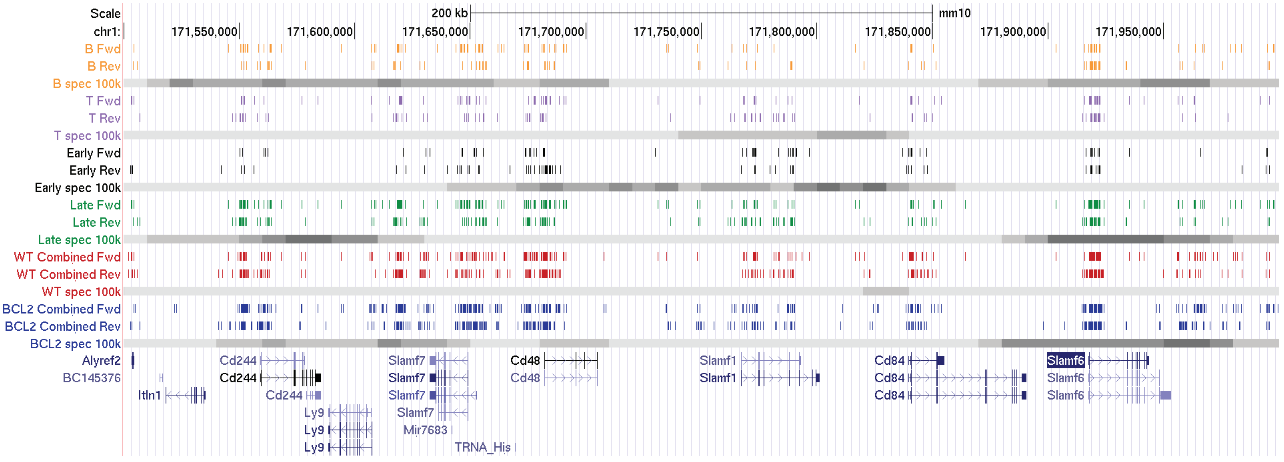

e chr6:48,600,000-48,800,000 - Gimap gene cluster

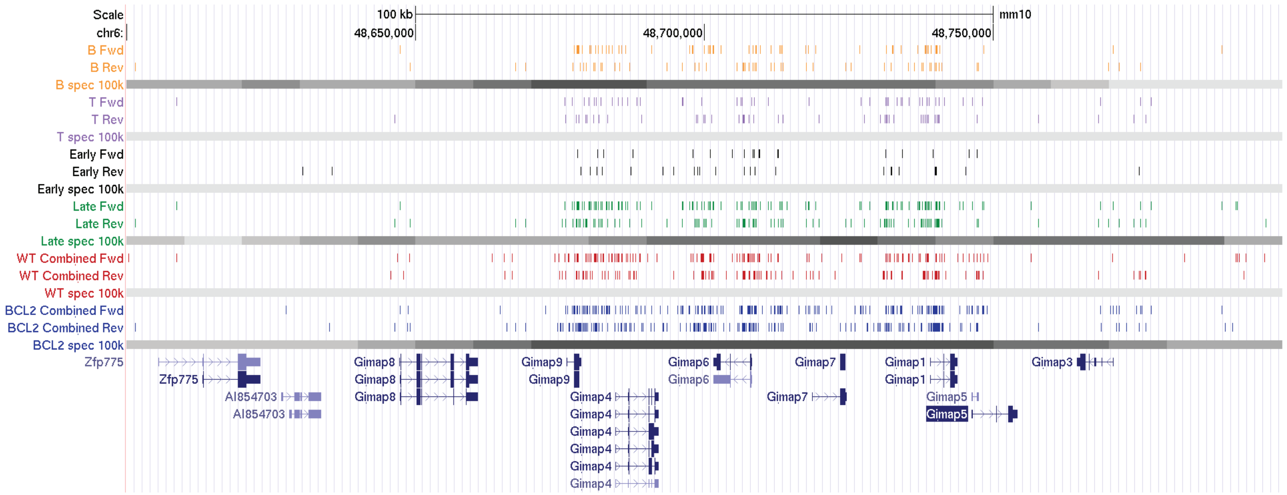

f chr10:38,600000-46,000000 - Prdm1 6q21 deleted region

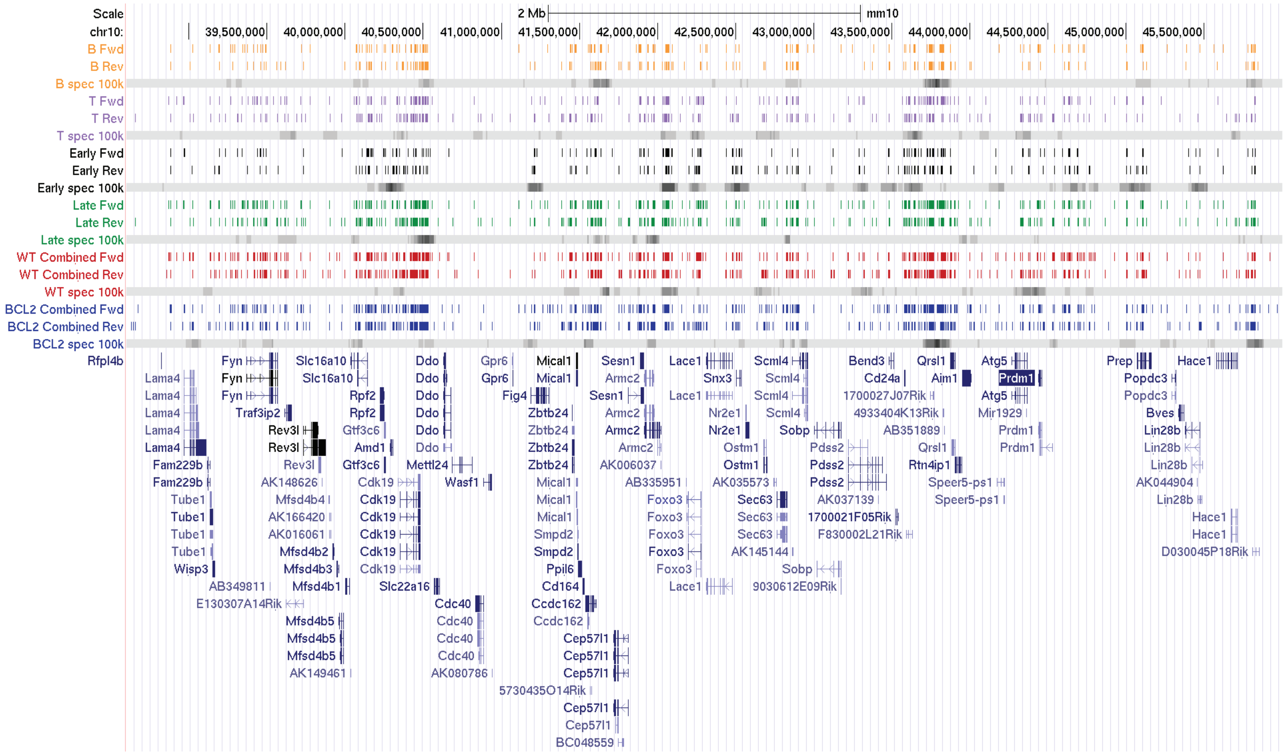

g chr7:25050000-25150000 - Pou2f2

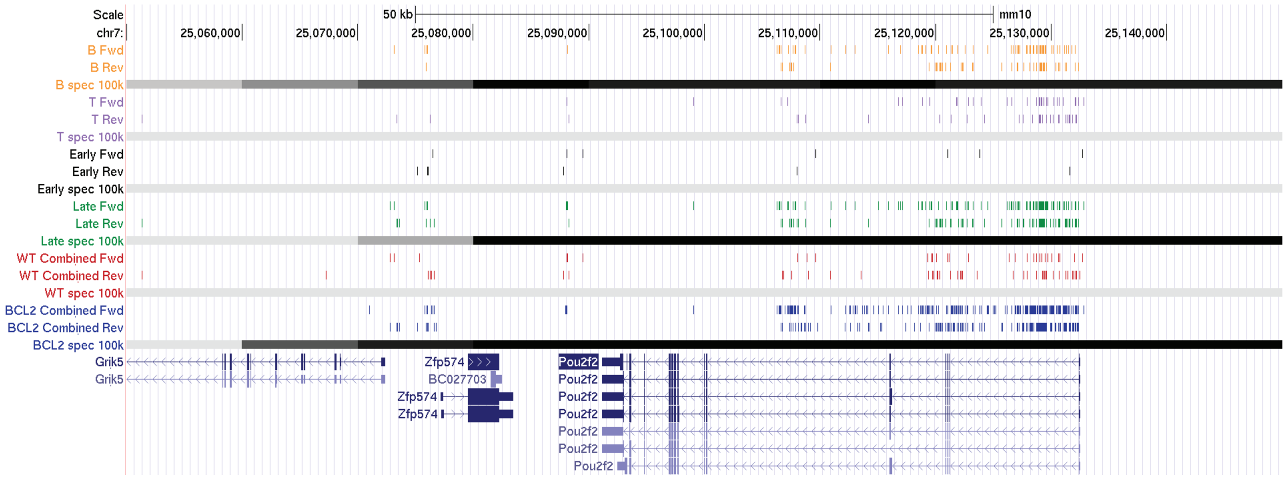

h chr11:11650000-11780000 - Ikzf1

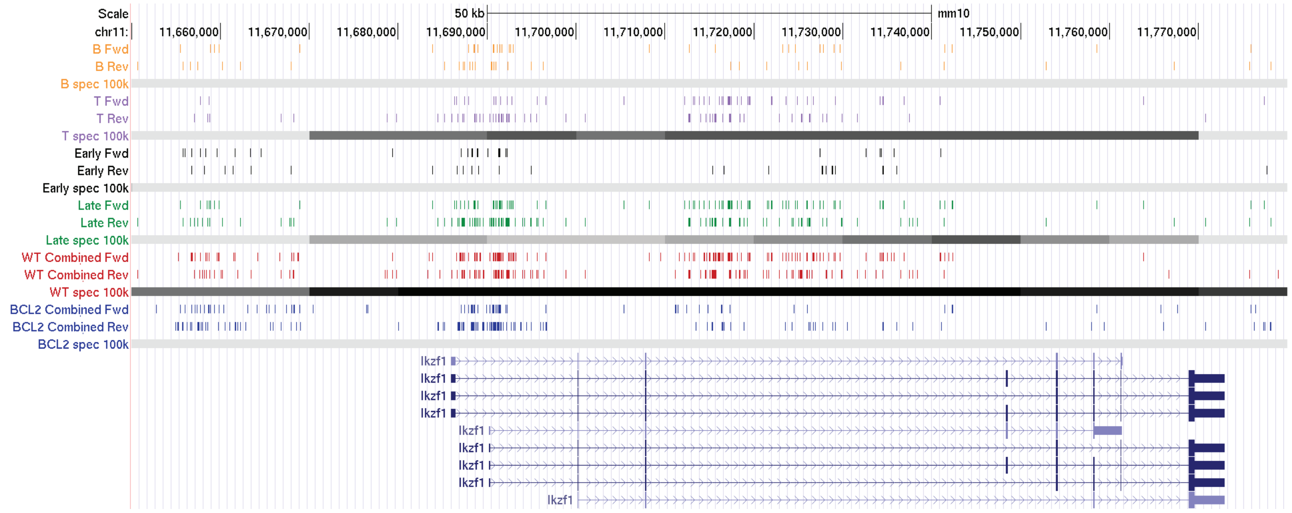

i chr11:98,440,000-98,560,000 - Ikzf3

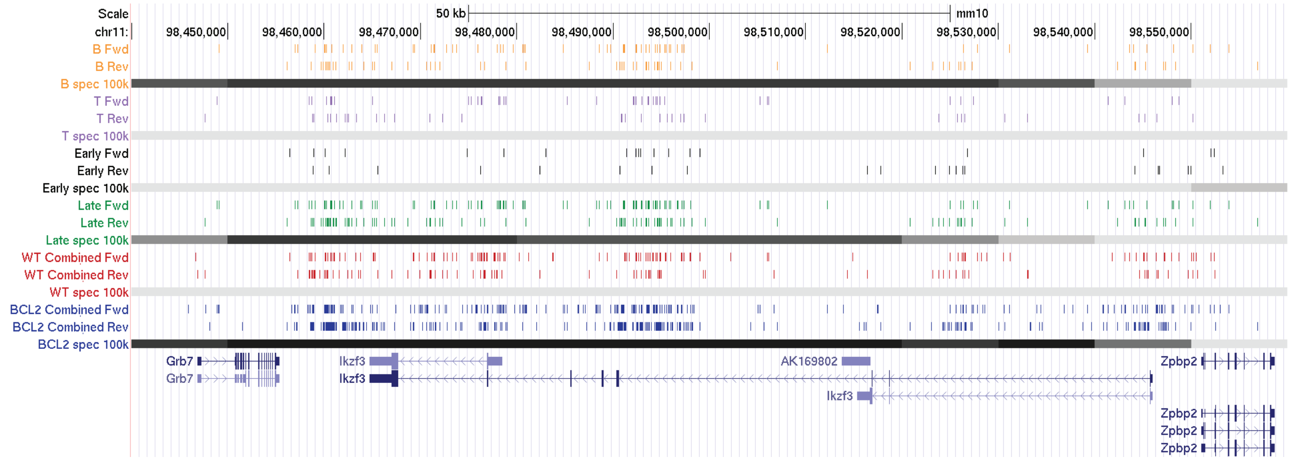

j chr11:44,570,000-45,070,000 - Ebf1

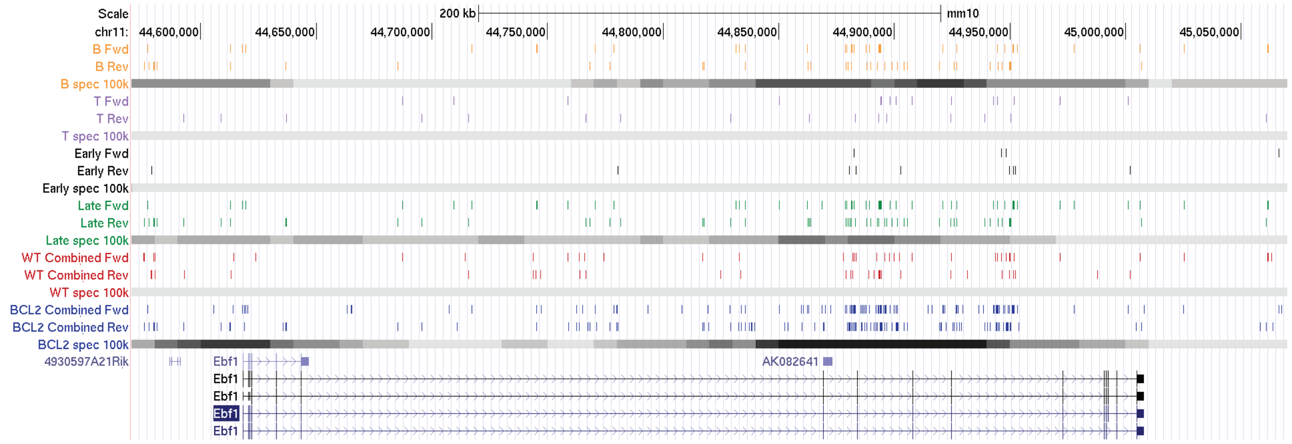

k chr18:35,800,000-35,950,000 - Cxxc5

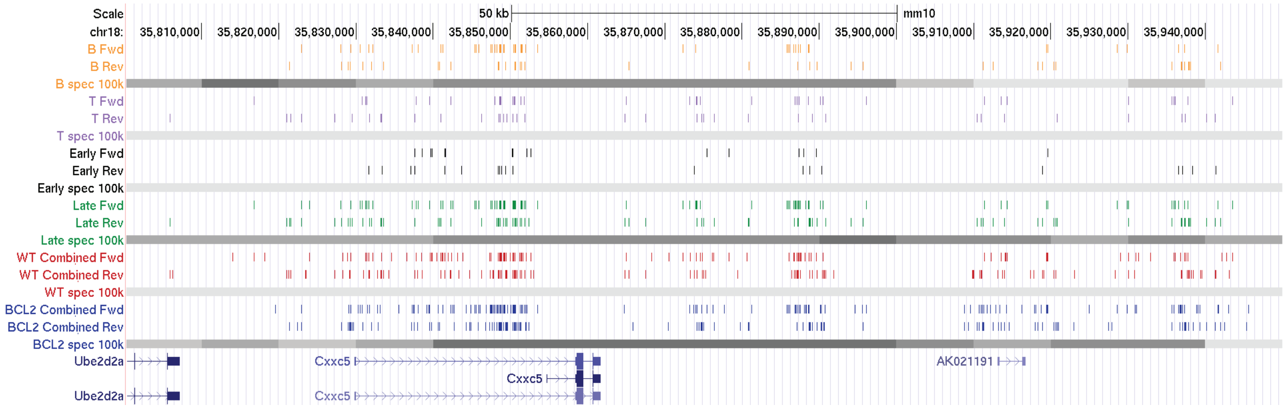

l chr16:36,550,00-36,750,000 - Cd86/Ildr1

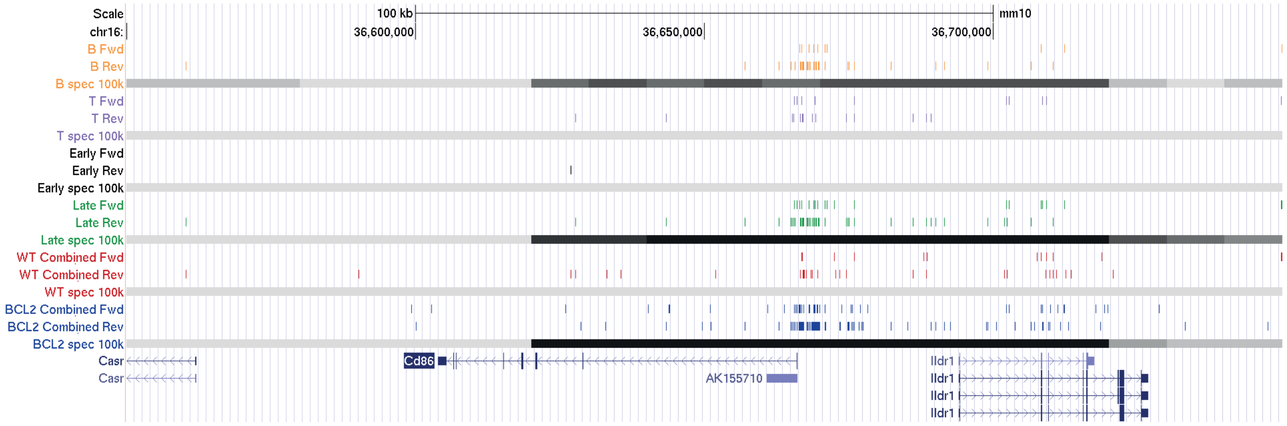

m chr:1:60,650,000-61,100,000 - Cd28/Ctla4/Icos

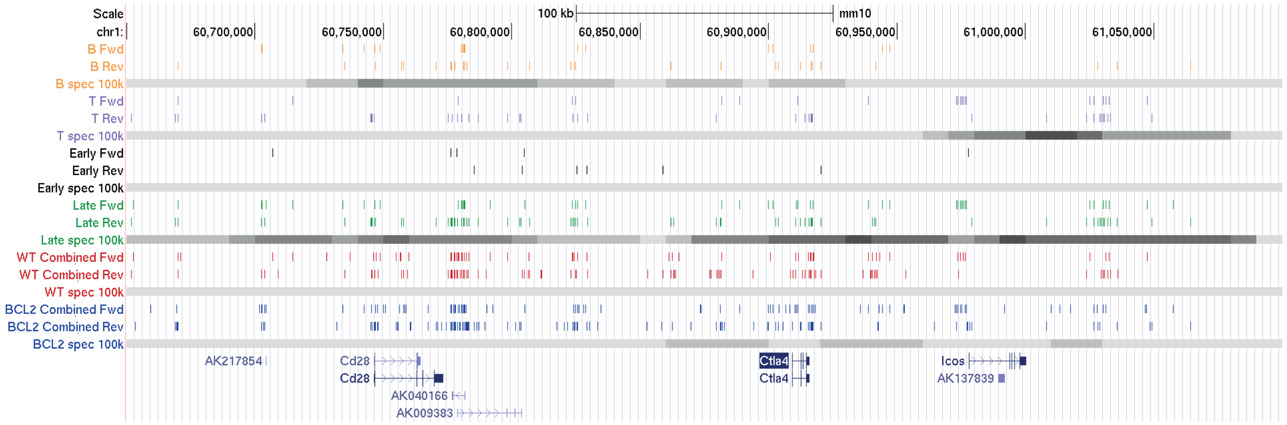

n chr:10:78,000,000-78,200,000 - Icosl

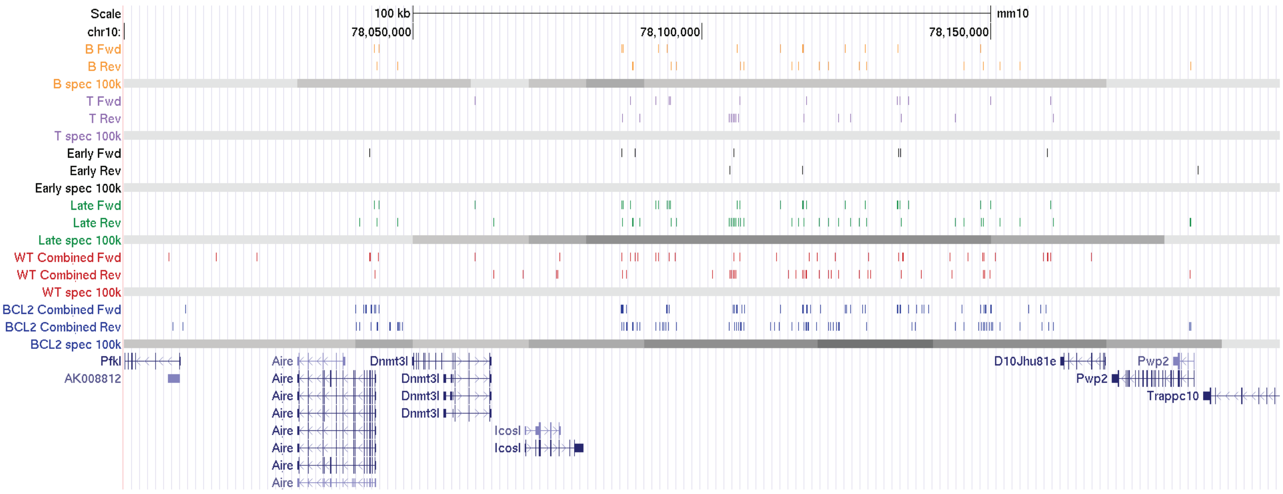

o chr:19:29,350,000-29,550,000 - Cd274/Pdcd1lg2

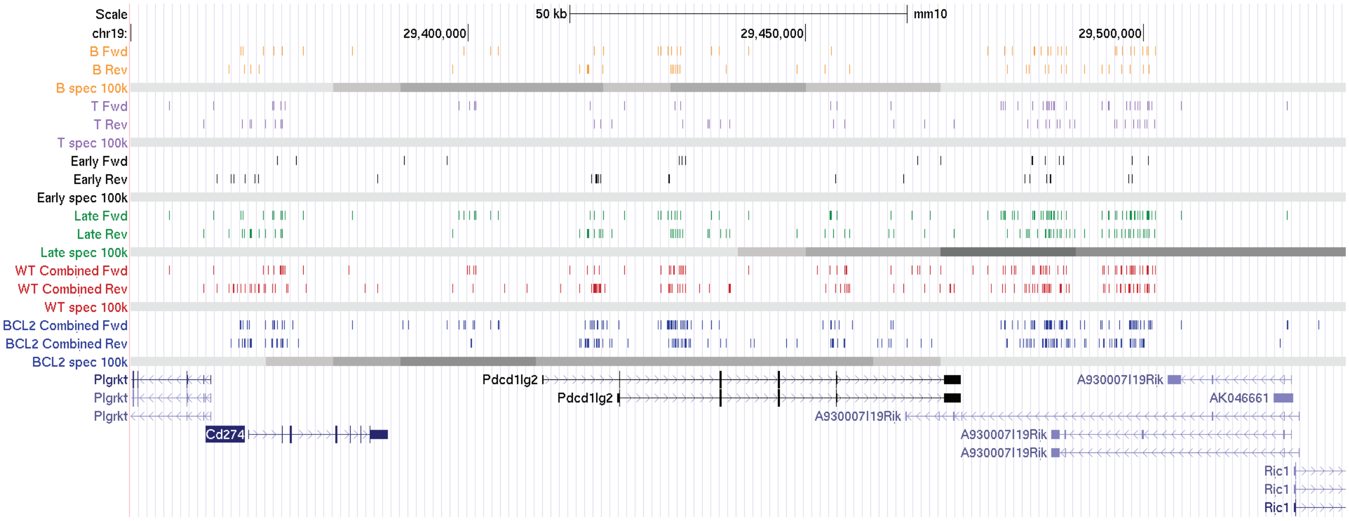

p chr:1:93,980,000-94,100,000 - Pdcd1

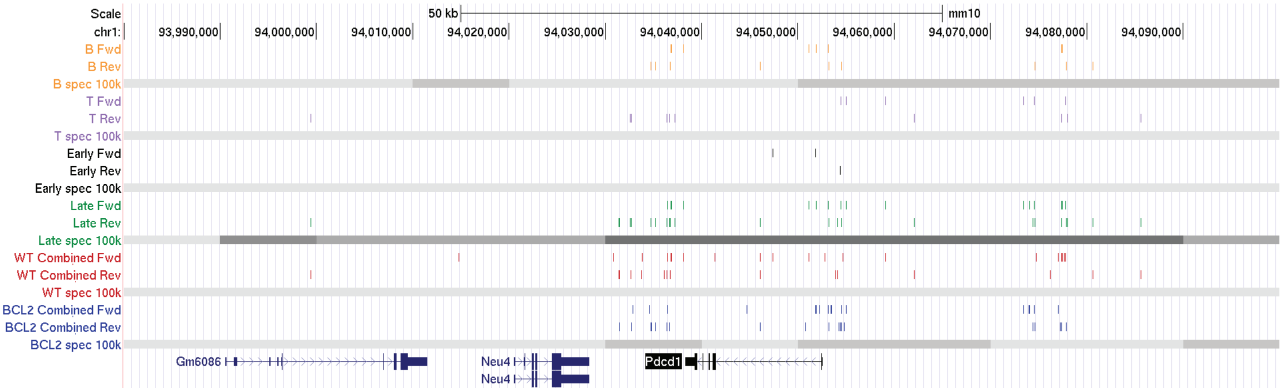

q chr17:33,883,511-36,364,198 - MHC loci

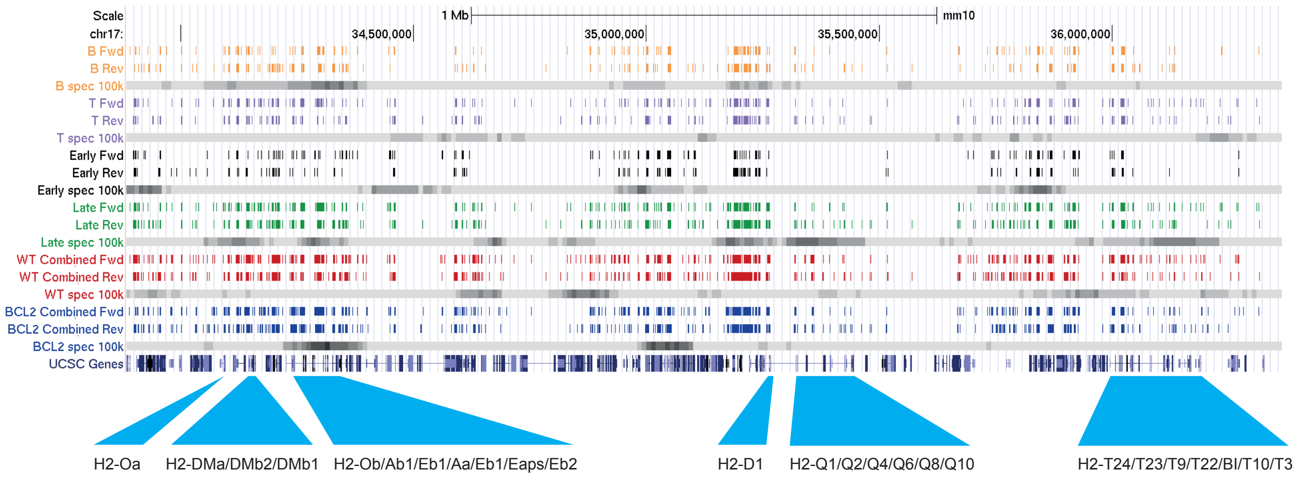

r chr9:178900000-17960000 - Smyd3

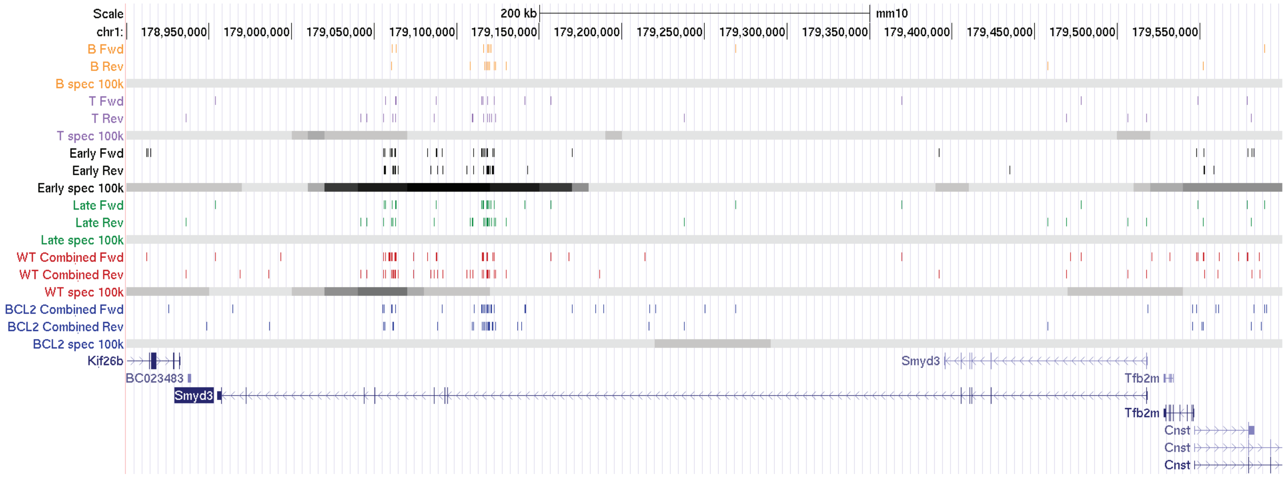

Supplementary Figure 11 – Example loci displaying selection

Distributions of integrations and selection tracks over various regions of the genome discussed in the main text. Each colored row of vertical lines represents the forward and reverse strand integrations of each category of mice. Grey bands below each colored row represent the level of selection evidenced by Fisher's exact tests. a) The entire chromosome 15 is represented. The region showing the most significant selection highlighted in yellow corresponds to the *Myc/Pvt1/Gsdmc* family loci. Other loci displayed b) *Myc/Pvt1/Gsdmc family* c) *Rel/Papolg/Bcl11a* d) *Slamf* gene cluster e) *Gimap* gene cluster f) the 6p21 region deleted in DLBCL containing *Prdm1* g) *Pou2f2* h) *Ikzf1* i) *Ikzf3* j) *Ebf1* k) *Cxxc5* l) *Cd86/Ildr1* m) *Cd28/Ctla4/Icos* n) *Icosl* o) *Cd274/Pdcd1lg2* p) *Pdcd1* q) the MHC loci of chromosome 17 r) *Smyd3*.

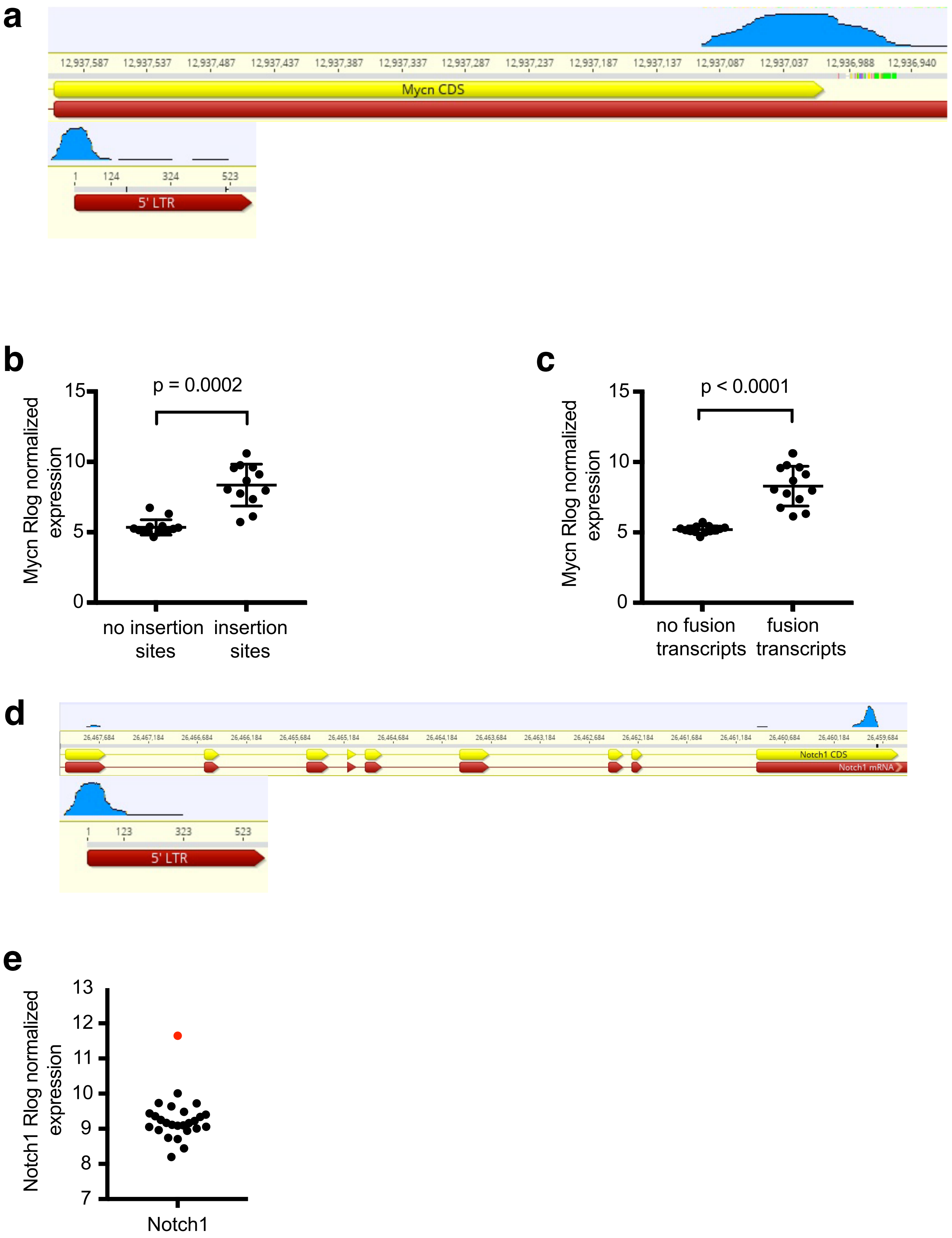
a
12,937,587
12,937,537
12,937,487
12,937,437
12,937,387
12,937,337
12,937,287
12,937,237
12,937,187
12,937,137
12,937,087
12,937,037
12,936,988
12,936,940
Mycn CDS
1
124
324
523
5' LTR
b
p = 0.0002
Mycn Rlog normalized expression
15
10
5
0
no insertion sites
insertion sites
c
p < 0.0001
Mycn Rlog normalized expression
15
10
5
0
no fusion transcripts
fusion transcripts
d
26,467,684
26,467,184
26,466,684
26,466,184
26,465,684
26,465,184
26,464,684
26,464,184
26,463,684
26,463,184
26,462,684
26,462,184
26,461,684
26,461,184
26,460,684
26,460,184
26,459,684
Notch1 CDS
Notch1 mRNA
1
123
323
523
5' LTR
e
Notch1 Rlog normalized expression
13
12
11
10
9
8
7
Notch1

Supplementary Figure 12 – Fusion transcripts identified by RNAseq of MuLV tumors.

a) *Mycn* fusion transcripts mapped to the *Mycn* locus and the 5' LTR of MuLV. Nearly all chimeric RNA reads were found to have corresponding inserts, however no integrations with NC <0.09 were detected in the transcriptome (a single clonal NC 0.75 antisense insertion in the region had no corresponding RNA reads). b&c) Mycn integrations significantly up regulate expression whether the presence of integrations is classified by DNA or RNA. Mean and +/-1SD are indicated. d) Notch1 fusion transcripts mapped to the Notch1 locus and the 5' LTR of MuLV. Two fusion transcripts were identified in Notch1. One highly expressed fusion (58 junction spanning reads) corresponding to the most clonal integration within the final exon causing replacing the final 73 AA of Notch1 with a 5 AA extension encoded by the 5' LTR, thereby deleting a "domain of unknown function" (DUF3454 pfam11936) conserved in Notch family members including the WSSSSP motif. A lower expressed transcript with an intronic junction (4 junction spanning reads) 3' of exon 26 permits a cryptic splice donor from the intron (CTG|GAGGTG) to splice into the MuLV genome at bp 8267, removing the last 869AA and truncating the protein just after the first cleavage site between the NOD (IPR0106660) and NOPD (IPR011656) domains, thus mimicking a cleaved extracellular domain. Equivalent truncations have been previously identified in MuLV and human lymphoid malignancies. Corresponding clonal inserts were also found in the DNA of this sample. e) The Notch1 inserted tumor that had the highest Notch1 expression of 26 samples is indicated in red.

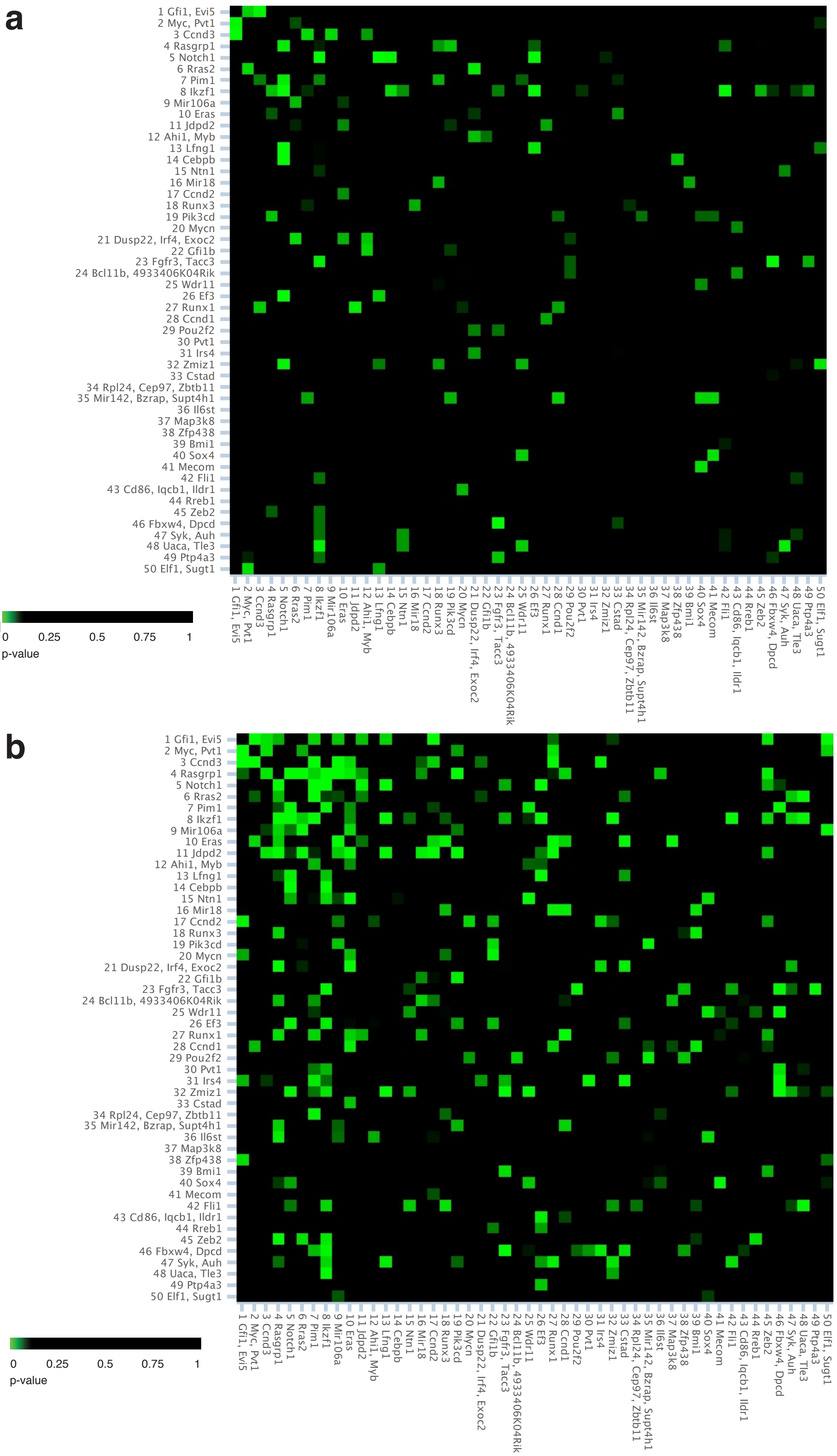
a
1 Gfi1, Evi5
2 Myc, Pvt1
3 Ccnd3
4 Rasgrp1
5 Notch1
6 Rras2
7 Pim1
8 Ikzf1
9 Mir106a
10 Eras
11 Jdpd2
12 Ahi1, Myb
13 Lfng1
14 Cebpb
15 Ntn1
16 Mir18
17 Ccnd2
18 Runx3
19 Pik3cd
20 Mycn
21 Dusp22, Irf4, Exoc2
22 Gfi1b
23 Fgfr3, Tacc3
24 Bcl11b, 4933406K04Rik
25 Wdr11
26 Ef3
27 Runx1
28 Ccnd1
29 Pou2f2
30 Pvt1
31 Irs4
32 Zmiz1
33 Cstad
34 Rpl24, Cep97, Zbtb11
35 Mir142, Bzrap, Supt4h1
36 Il6st
37 Map3k8
38 Zfp438
39 Bmi1
40 Sox4
41 Mecom
42 Fli1
43 Cd86, Iqcb1, Ildr1
44 Rreb1
45 Zeb2
46 Fbxw4, Dpcd
47 Syk, Auh
48 Uaca, Tle3
49 Ptp4a3
50 Elf1, Sugt1
0
0.25
0.5
0.75
1
p-value
b
1 Gfi1, Evi5
2 Myc, Pvt1
3 Ccnd3
4 Rasgrp1
5 Notch1
6 Rras2
7 Pim1
8 Ikzf1
9 Mir106a
10 Eras
11 Jdpd2
12 Ahi1, Myb
13 Lfng1
14 Cebpb
15 Ntn1
16 Mir18
17 Ccnd2
18 Runx3
19 Pik3cd
20 Mycn
21 Dusp22, Irf4, Exoc2
22 Gfi1b
23 Fgfr3, Tacc3
24 Bcl11b, 4933406K04Rik
25 Wdr11
26 Ef3
27 Runx1
28 Ccnd1
29 Pou2f2
30 Pvt1
31 Irs4
32 Zmiz1
33 Cstad
34 Rpl24, Cep97, Zbtb11
35 Mir142, Bzrap, Supt4h1
36 Il6st
37 Map3k8
38 Zfp438
39 Bmi1
40 Sox4
41 Mecom
42 Fli1
43 Cd86, Iqcb1, Ildr1
44 Rreb1
45 Zeb2
46 Fbxw4, Dpcd
47 Syk, Auh
48 Uaca, Tle3
49 Ptp4a3
50 Elf1, Sugt1
0
0.25
0.5
0.75
1
p-value

Supplementary Figure 13 – Co-mutation analyses

Co-mutation profiles of the top 50 GKC CIS loci from clonal integrations. The p-value of Fisher's exact tests is represented by the green color intensity and indicates mutations at both loci occur more frequently than expected by chance. a) Analysis was performed using only clonal integrations from late stage lymphomas. b) Analysis was performed using clonal integrations to categorize samples into two groups and then counting the frequencies of all mutations (both clonal and subclonal) at other loci in those two groups. The second analysis identifies many co-mutated gene pairs that would not be identified using only clonal integrations.

Time after injection	Cohort 1				Cohort 2				Cohort 3				Total virus infected animals			
	Virus Infected		Uninfected Control		Virus Infected		Uninfected Control		Virus Infected		Uninfected Control					
	WT C57BL/6	Eμ-*BCL2* C57BL/6	WT C57BL/6	Eμ-*BCL2* C57BL/6	WT F1	Eμ-BCL2 F1	WT F1	Eμ-BCL2 F1	WT F1	VavP-*BCL2* F1	WT F1	VavP-*BCL2* F1	WT	Eu-*BCL2*	VavP-*BCL2*	All BCL2
**Day 9**	1	3	7	1	0	0	5	7	0	6	4	2	1	3	6	9
**Day 14**	4	3	4	2	7	3	5	3	0	0	0	0	11	6	0	6
**Day 28**	0	7	3	4	4	4	5	5	2	3	5	2	6	11	3	14
**Day 42**	3	4	3	2	5	4	7	5	4	6	8	1	12	8	6	14
**Day 56**	2	4	2	4	5	4	5	5	5	5	2	4	12	8	5	13
**Day 84**	3	5	4	3	0	0	0	0	8	10	0	0	11	5	10	15
**Day 112**	4	3	4	3	0	0	0	0	8	2	4	6	12	3	2	5

Supplementary Table 1

Table of ages and genotype of animals included in the time course analysis.

loci within CNAs	loci outside CNAs	bases within CNAs	bases outside CNAs	2-tailed p-value	right handed p-value	left handed p-value	Cohort	Method	Study	Reference
79	194	80428683	267771836	0.026	0.015	0.990	48 cases DLBCL	GISTIC2	gdac.broadinstitute.org	http://dx.doi.org/10.7908/C1HQ3Z31
6	267	3040112	345160406	0.034	0.034	0.989	180 cases DLBCL	GISTIC	Monti et al.	http://dx.doi.org/10.1016/j.ccr.2012.07.014
25	248	18271599	329928920	0.009	0.005	0.997	64 cases FL	GISTIC	Orrichio et al.	http://dx.doi.org/10.1084/jem.20132120
44	229	41607271	306593248	0.040	0.025	0.984	198 cases FL	GISTIC	Bouska et al.	http://dx.doi.org/10.1182/blood-2013-05-500595
9	264	6743418	341457100	0.117	0.086	0.958	609 cases DLBCL	GISTIC	Green et al	http://dx.doi.org/10.1038/ncomms4904

Supplementary Table 2

Using a non-redundant list of 278 selected regions identified in this screen by any criteria, 273 were unambiguously mappable to orthologous region on Hg19. Coordinates of recurrent copy number aberrations (CNAs) from 5 studies of human mature B cell lymhpoma were mapped to Hg19 as required. By Fisher's exact test there is significant overlap between the 273 loci and the recurrent copy number aberrations of 4 of the 5 human datasets.
