# Supplementary material for: Subclonal mutation selection in mouse lymphomagenesis identifies known cancer loci and suggests novel candidates
Source: Nat Commun. 2018 Jul 9;9:2649. doi: 10.1038/s41467-018-05069-9 (PMC6037733; doi:10.1038/s41467-018-05069-9)
Supplement: Supplementary file 3 — Description of Additional Supplementary Files [file 41467_2018_5069_MOESM3_ESM.pdf]

Description of Additional Supplementary Files

File Name: Supplementary Data 1
Description: **DTW vs KS Comparison.** Clustering into two groups is compared using both Dynamic Time Warp (DTW) and Kolmogorov Smirnov (KS) as distance measures. 19 of 518 samples are allocated to different clusters when comparing the two methods. These samples assigned to different clusters had entropy values over a narrow range between 3.451 and 3.692.

File Name: Supplementary Data 2
Description: **GKC CIS Selection.** Limiting analysis to late stage clonal integrations GKC identifies 311 CIS loci with a p-value

File Name: Supplementary Data 3
Description: **Genome wide scan for selection.** Genome wide scanning of subclonal mutation selection. A scanning 100kb window is moved across the genome in increments of 10kb. For each window the number of insertions in each class (early/late, forward strand/reverse strand, BCL2 transgenic/wild type, B cell/T cell) is counted and the likelihood of this distribution between groups is estimated using Fisher's exact test. By comparing neighboring windows p-value minima are identified (i.e. windows where the p-value is higher on either side). If a run of 2 or more minima are less than 100,000bp from each other all but the lowest minima will be discarded. For each of the remaining local p-value minima, to assign gene names the nearest peak identified by Gaussian kernel convolution (using the CIMPL/KCRBM packages) and the genes associated with this peak are identified. Each locus with a false discovery rate

File Name: Supplementary Data 4
Description: **Strand bias in early & late stage samples.** Comparison of strand bias in early vs. late stage cohorts using equal numbers of integrations. The total number of early stage integrations is 81316. A subset of late 81316 stage integrations was randomly selected and regions with strand/orientation bias were identified using 100kb scanning windows across the entire genome. 16 late-stage loci were significant (FDR < 0.05) after correcting for multiple testing but no early stage loci were found to be significant.

File Name: Supplementary Data 5
Description: **Candidate genes.** Combined list of all candidate genes implicated by one or more criteria in the genome wide scans of subclonal mutation selection.

File Name: Supplementary Data 6
Description: **Cancer census overlap.** Candidates implicated by subclonal selection correspond to genes from the cancer genome census. 27 of these genes map within 200kb of a clonal GKC CIS (pvalue < 312). The distance of subclonal selection peaks from GKC CIS is indictated. Peaks not within 200kb of significant clonal GKC CIS are highlighted. Adjacent pairs of genes implicated by inserts at the same locus are shaded (Kit & Kdr, Bcl6 & Lpp, Stat5b & Stat3, Rel & Bcl11a).

File Name: Supplementary Data 7
Description: **Exome mutation enrichment.** Mutation counts from 12 studies of hematologic malignancies were downloaded from the cBio repository (cBioportal.org). Two lists of orthologues of candidate genes (a KCRBM automated list and a list that includes additional curated candidates) were used to find overlap between coding mutations in orthologous human genes. Significance of overlap with between candidate lists and each set of mutated genes (all genes, genes mutated more

than once, genes mutated once) is calculated by fisher's exact test using all protein coding genes and tabulated for all comparisons.

File Name: Supplementary Data 8
Description: **Supporting Literature.** Supporting literature implicating a role for candidate genes in hematologic malignancies with an emphasis on lymphoid malignancies. Cancer genome census genes and Intogen cancer drivers are also listed.

File Name: Supplementary Data 9
Description: **Fusion Transcript Counts.** Insertion clonality and fusion transcript read counts are tabulated for Mycn & Notch1 in the 26 samples sequenced. Selected RNAs (each estimated to have between 65% - 75% B cells by flow cytometry) were subject to RNA sequencing (stranded ribosomal RNA depleted). Chimeric genome/virus transcripts were identified using the Virusfinder package (55). Selected loci were then remapped and visually inspected in order to verify these sites correspond to true chimeric transcripts. Globally only a tiny fraction of read pairs were found to be chimeric, suggesting chimeric transcripts within MuLV lymphomas are only responsible for selection of retroviral integrations in a fraction of the clonal loci. Surprisingly, no chimeric reads were found within Ikzf1, a known tumor suppressor gene with frequent intragenic integrations in MuLV lymphomas, suggesting any fusion transcripts may be unstable and or these viruses result in silencing of the locus.

File Name: Supplementary Data 10
Description: **GWAS study enrichment.** Genome wide association studies of acute lymphoblastic leukemia (ALL), diffuse large B cell lymphoma (DLBCL) and follicular lymphoma (FL) were taken from the literature and all SNPs significantly associated with disease are listed. Each single SNP or cluster of disease associated SNPs is mapped to the mouse genome mm10 using liftover (UCSC) and the nearest peaks are identified for the mapped mouse coordinates. CIS peaks within 200kb of a mapped over SNP coordinate are highlighted.

File Name: Supplementary Data 11
Description: **Co-mutation by exact tests.** Contingency table tests were performed on the top 50 clonal GKC CIS loci, the top 100 GKC CIS loci or by comparing the top 100 loci to the top 1155 loci. The number of tests that survive multiple testing correction (false discovery rate <0.05 or <0.1 calculated using the p.adjust() function in R) is indicated for the entire cohort of late stage lymphomas and for each of the four genotypes individually. The f.d.r. values were calculated for 100kb windows surrounding the top 50 vs top 50 clonal CIS loci, top 100 vs top 100, and top 100 vs all 1155 loci. Limiting the analysis to clonal mutations substantially reduces the power of each test and hence reduces the number of tests that survive multiple testing correction. Actual f.d.r. rates for each window pair are reported for the 100 x 1155 tests in the subsequent tab.
